# Cytology of the minor-vein phloem in 320 species from the subclass Asteridae suggests a high diversity of phloem-loading modes^†^

**DOI:** 10.3389/fpls.2013.00312

**Published:** 2013-08-21

**Authors:** Denis R. Batashev, Marina V. Pakhomova, Anna V. Razumovskaya, Olga V. Voitsekhovskaja, Yuri V. Gamalei

**Affiliations:** Laboratory of Plant Ecological Physiology, Komarov Botanical Institute, Russian Academy of SciencesSt. Petersburg, Russia

**Keywords:** phloem loading, companion cells, minor veins, symplast, apoplast, Asteridae

## Abstract

The discovery of abundant plasmodesmata at the bundle sheath/phloem interface in Oleaceae (Gamalei, 1974) and Cucurbitaceae (Turgeon et al., 1975) raised the questions as to whether these plasmodesmata are functional in phloem loading and how widespread symplasmic loading would be. Analysis of over 800 dicot species allowed the definition of “open” and “closed” types of the minor vein phloem depending on the abundance of plasmodesmata between companion cells and bundle sheath (Gamalei, 1989, 1990). These types corresponded to potential symplasmic and apoplasmic phloem loaders, respectively; however, this definition covered a spectrum of diverse structures of phloem endings. Here, a review of detailed cytological analyses of minor veins in 320 species from the subclass Asteridae is presented, including data on companion cell types and their combinations which have not been reported previously. The percentage of Asteridae species with “open” minor vein cytology which also contain sieve-element-companion cell complexes with “closed” cytology, i.e., that show specialization for both symplasmic and apoplasmic phloem loading, was determined. Along with recent data confirming the dissimilar functional specialization of structurally different parts of minor vein phloem in the stachyose-translocating species *Alonsoa meridionalis* (Voitsekhovskaja et al., 2009), these findings suggest that apoplasmic loading is indispensable in a large group of species previously classified as putative symplasmic loaders. Altogether, this study provides formal classifications of companion cells and of minor veins, respectively, in 24 families of the Asteridae based on their structural features, opening the way to a close investigation of the relationship between structure and function in phloem loading.

## Introduction

Since their discovery in 1879 by Tangl (1879), plasmodesmata have been assumed to serve the intercellular transport of metabolites in plants, and the development of numerous plasmodesmal connections was considered an indicator of intensified symplasmic exchange between the connected cells (Tyree, 1970). The highest densities of plasmodesmata per cell surface unit occur in minor veins of leaves at the interface between bundle sheath cells and companion cells belonging to the “intermediary cell” type, as are found in some dicot families, e.g., Cucurbitaceae and Oleaceae (Gamalei, 1974; Turgeon et al., 1975). The walls between intermediary cells (ICs) and bundle sheath cells in *Fraxinus ornus* minor veins contain about 60 plasmodesmata per μm^2^ surface facing the bundle sheath, and up to 140 plasmodesmata per μm^2^ surface on the IC side (Gamalei, 1990, 1991). These extremely high densities of symplasmic connections were the reason why species with ICs in minor vein phloem were suggested to load assimilates into the phloem preferentially via the symplast. In contrast, species containing in their minor vein phloem either “ordinary” companion cells (OCs) with only few plasmodesmata at the bundle sheath side (about 1 per μm^2^; Gamalei, 1991), or transfer companion cells (TCs) characterized by even lower plasmodesmal density (about 0.1 per μm^2^; Gamalei, 1991) and cell wall ingrowths, were considered apoplasmic phloem loaders.

The cytology of companion cells in leaf minor veins of dicotyledonous plants, often in relation with the mode of phloem loading, was subject of extensive studies covering a large number of species (e.g., Pate and Gunning, 1969; Peterson and Yeung, 1975; Turgeon et al., 1975, 1993; Madore et al., 1986; Fisher, 1986, 1991; Schmitz et al., 1987; Gamalei, 1989, 1990, 1991; van Bel and Gamalei, 1992; van Bel et al., 1992; Kempers et al., 1998; Haritatos et al., 2000; Goggin et al., 2001; Turgeon et al., 2001; Turgeon and Medville, 2004; Reidel et al., 2009). The most comprehensive analysis, however, has been performed by Gamalei encompassing over 800 species from over 140 families (Gamalei, 1990). He described the extremes of minor vein organization, type 1 and 2, which differed both in the pattern of the first divisions of the phloem initial during vein development, and in the abundance of symplasmic connections between companion cells and bundle sheath (Gamalei, 1989, 1991). The first division of the phloem initial was anticlinal in type 1 species but periclinal in type 2 species which resulted in different spatial organization of mature minor veins of type 1 and 2 species, respectively. The position of the first division plane showed a striking correlation with the abundance of plasmodesmata at the companion cell side facing the bundle sheath. This allowed designating types 1 and 2 minor veins as “open” and “closed,” respectively (Gamalei, 1989). Minor veins of type 2 could be divided into 2a, 2b, and 2c; 2a species contained companion cells without cell wall ingrowths, 2b species contained TCs with cell wall ingrowths, and the 2c group comprised species with Kranz anatomy. It was noticed that type 1 species are often represented by trees or woody shrubs while type 2 species are mostly herbs (Gamalei, 1989). A large group of species designated 1-2a showed numbers of plasmodesmata at the companion cell/bundle sheath boundary intermediate between 1 and 2a, and minor veins without well-defined spatial organization. For this group, growth form and phloem evolution were not clear (Gamalei, 1989).

This classification was modified with the focus on the number of symplasmic connections at the mesophyll/phloem interface (Gamalei, 1991). Four types were distinguished, two “open” ones (1 and 1-2a) and two “closed” ones (2a and 2b); it should be kept in mind that also in this classification, the first numeral 1 or 2 carries information on the degree of symplasmic continuity between mesophyll and phloem as well as on the ontogenesis of the minor vein phloem. Type 1 corresponded to species with numbers of plasmodesmata per square μm cell surface between 100 and 10, type 1-2a included species with these numbers between 10 and 1, types 2a and 2b designated plants with plasmodesmata numbers per square μm cell surface below 1, whereas type 2b specified species with TCs where cell wall ingrowths increase apoplasmic transport (Gamalei, 1991). The introduction of these four types has been very helpful for elucidating the evolution of phloem loading mechanisms (Turgeon et al., 2001; Rennie and Turgeon, 2009) as well as for functional studies (e.g., Turgeon et al., 1993; Hoffmann-Thoma et al., 2001; Turgeon and Medville, 2004). Indeed, functional tests on a range of species in the studies of van Bel et al. using the classification of Gamalei (van Bel et al., 1992) provided compelling evidence for symplasmic phloem loading which was strongly disputed before. At the same time, many plants contain companion cells of more than one structural type in their minor veins, a fact which renders them beyond the scope of a division into “open” and “closed” types and becomes important when analyzing phloem loading mechanisms and their relative contribution to phloem transport in a given species. An example is *Alonsoa meridionalis*, a type 1 species, which contains ICs in its minor veins and was originally classified as a putative symplasmic loader but was shown to perform also apoplasmic sucrose loading in a second type of companion cells present in its minor veins, the OC (Voitsekhovskaja et al., 2009). Thus, a comprehensive view of a phloem ending as a functional unit can rely only on the cytology of all cells involved in assimilate loading. However, the possibility of different structural types of sieve element—companion cell complexes (SE-CCCs) to occur in minor veins of one and the same species is neglected in the four-types-scheme which limits its usefulness and may even render it misleading from the functional point of view in some cases.

In the present study, the cytology of minor veins was examined in a number of species from the subclass Asteridae (Moore et al., 2010), one of the major clades of the core eudicots (for the list of species see Supplemental Table 1). For the abovementioned reasons, we paid attention to the ultrastructure of all cells in minor vein phloem of the species investigated, as well as to the spatial organization of the minor vein. This study, together with data from published sources (Pate and Gunning, 1969; Peterson and Yeung, 1975; Evert, 1980; Madore and Grodzinski, 1984; Madore et al., 1986; Ding et al., 1988; McCauley and Evert, 1989; Fisher, 1991; van Bel et al., 1992; Turgeon et al., 1993; Roberts et al., 1997; Batashev and Gamalei, 2000, 2005; Voitsekhovskaja et al., 2006; Gamalei et al., 2008; Reidel et al., 2009), resulted in the analysis of 320 species belonging to 200 genera. This makes the Asteridae the best investigated subclass of dicots to date in terms of organization of phloem endings and structure of companion cells. Here, we give an overview of companion cell types in Asteridae and describe some structures not reported previously. We describe the ways how these cells can be combined in a phloem ending. We show that in the overwhelming number of species, spatial organization of minor veins and symplastic continuity between companion cells and bundle sheath are strictly correlated, confirming the conclusions made by Gamalei (1989). However, we also present a few striking exceptions to this rule indicating the need for further categories of minor veins. We also provide a formal classification of minor veins in Asteridae on the basis of their companion cell type(s), spatial organization and a few additional features which may be potentially important to understand the assimilate pathways during phloem loading. These data lay a foundation for phylogenetic and functional analyses of the phloem in Asteridae, leading to a deeper understanding of the role of plasmodesmata in phloem loading.

## Materials and methods

### Plant material

Plants were collected (1) from their native habitats during expeditions in Altai, Cola peninsula, Leningrad region, Magadan region; (2) from botanical gardens of the Komarov Botanical Institute RAS (St.-Petersburg, Russia), from the Nikitskiy botanical garden (Crimea, Ukraine) and from the botanical garden at Altai State University (Barnaul, Russia). (3) Tropical species were collected from greenhouses of the botanical garden of the Komarov Botanical Institute RAS, and several Apocynaceae species were collected in parks of Bangkok (Thailand). The list of species studied is shown in Supplemental Table 1. Altogether, 320 species from 24 families *in sensu* APGIII, 2009 (295 species originally studied in the laboratory of Yu. V. Gamalei plus 25 species from published studies from other laboratories) were included in this analysis.

### Transmission electron microscopy (TEM) studies

The ultrastructure of the minor vein phloem (6–7 transverse sections of veins of the highest orders) was studied by means of TEM. Mature fully expanded leaves (3–4 leaves from different plants for every species studied) were analyzed. Leaf pieces (3 × 4 mm) were infiltrated with cold fixative (3% glutaraldehyde, 3% sucrose in 0.1 M potassium phosphate buffer pH 7.2), incubated in fresh fixative for 6 h and washed in buffer six times for 10 min each. The material was then post-fixed for 16 h in 2% osmium tetroxide in potassium phosphate buffer at 4°C, dehydrated in 30% and 50% ethanol for 20 min each, contrasted with 1.5% uranyl acetate in 70% ethanol for 2 h, further dehydrated in an ethanol:acetone series and embedded in Epon-Araldite epoxy resin. Ultrathin sections (40–60 nm) were cut with glass knives on an LKB-III microtome (LKB, Stockholm, Sweden), contrasted on grids using 2% lead citrate, and viewed and photographed at 75 kV with a Hitachi H-600 electron microscope (Tokyo, Japan).

### Sugar analysis

Leaves of *Allamanda cathartica* L., *Alstonia macrophylla* Wall. et G. Don, *Plumelia rubra* L. and *Thevetia nereifolia* Juss. ex Steud. (Apocynaceae), and of *Melampyrum sylvaticum* L., *Euphrasia fennica* Kihlm. and *Rhinanthus minor* L. (Orobanchaceae) were extracted twice with 80% ethanol at 60°C. Seven hundred microliter of each extract (corresponding to approx. 500 mg fresh weight) were vacuum-dried at 40°C in a rotary evaporator (Buechi, Flawil, Switzerland) and subjected to derivatization in a mixture of (N,O-Bis-trimethylsilyl)-trifluoroacetamide:pyridine (1:1, v/v) (Sigma-Aldrich, Deisenhofen, Germany) in a hermetically closed tube for 15 min at 100°C. A gas chromatograph Agilent 6850 (Agilent Technologies, Santa Clara, CA, USA) equipped with a mass selective detector Agilent 5975C was used, supplied with a capillary column HP-5MS (30 m length, 0.25 mm diameter, 0.25 μm film thickness; J&W Scientific, Folsom, CA, USA). Helium was used as a carrier gas at a flow rate of 1.3 ml/min. The column was operated at an initial temperature of 70°C and adjusted to 320°C at 6°C/min. The temperature of the injector was 330°C by the split flow 50:1. The injected volume was 1 μl. The internal standard used was n-C_23_ hydrocarbon (Sigma-Aldrich, Deisenhofen, Germany). The data were collected and processed with the Agilent ChemStation system. Mass spectra were interpreted, and substances identified, with the AMDIS (Automated Mass-Spectral Deconvolution and Identification System) software using NIST 2008 and Wiley6 libraries. Quantification of chromatograms was performed using UNICHROM software (New Analytical Systems, Minsk, Belarus).

## Results

### Cytological diversity of companion cells in asteridae species

Since companion cells are key players in phloem loading, we first focused on their structural diversity in minor veins of Asteridae. To provide a better overview of observed structures, we analyzed several features which were then used to build a classification. Three independent cytological characteristics of companion cells were selected: (1) presence vs. absence of plasmodesmal fields connecting companion cells to the bundle sheath; (2) presence vs. absence of cell wall ingrowths in companion cells; (3) type of plastids present in companion cells (leucoplasts vs. chloroplasts). Plasmodesmal fields are defined here as aggregations of plasmodesmata, either branched or simple, which can be clearly distinguished from the rest of the cell wall that may contain single plasmodesmata. We decided to use this characteristic as indicative for a considerable potential for symplasmic transport, in contrast to a few single plasmodesmata which are always present in any type of companion cell. Moreover, the distinction (plasmodesmal fields vs. single plasmodesmata) is qualitative and does not require elaborative counts of numbers of plasmodesmata per cell surface unit. The development of cell wall ingrowths increases the surface of metabolite exchange via apoplast. Thus, the first two characteristics indicate a high specialization level of companion cells adapted to symplasmic or apoplasmic phloem loading, respectively. The third feature, the type of plastids in companion cells, is easily distinguishable on micrographs. This feature has proved invaluable for discriminating between companion cells and parenchyma cells in minor veins (Russin and Evert, 1985; Reidel et al., 2009) but so far it has not been considered in relation to the phloem-loading mechanism. However, it should be taken into account that plastid retrograde signaling has been recently shown to be a potent regulator of plasmodesmata development (Burch-Smith et al., 2011). Also, the type of plastids in companion cells might be related to metabolic specialization of these cells. For instance, the fact that plastids in ICs are always leucoplasts and never chloroplasts might simply reflect the main function of these plastids as myo-inositol depots (Moore et al., 1997; Voitsekhovskaja et al., 2006), as the RFO synthesis by ICs requires high activities of myo-inositol production but not of sucrose production because sucrose is supplied by mesophyll cells.

We also included in the analyses two subordinate features, the mode of plasmodesmata branching in plasmodesmal fields and the morphology of cell wall protuberances. Asymmetric branching of plasmodesmata was shown to be an important diagnostic feature for ICs (Turgeon and Medville, 2004). Two different types of the morphology of cell wall protuberances have been described so far, reticulate and flange morphology (Offler et al., 2003); however, only reticulate protuberances were found in companion cells. Here, we describe as a novelty companion cells with cell wall ingrowths of flange morphology (see below).

**Figure 1:**
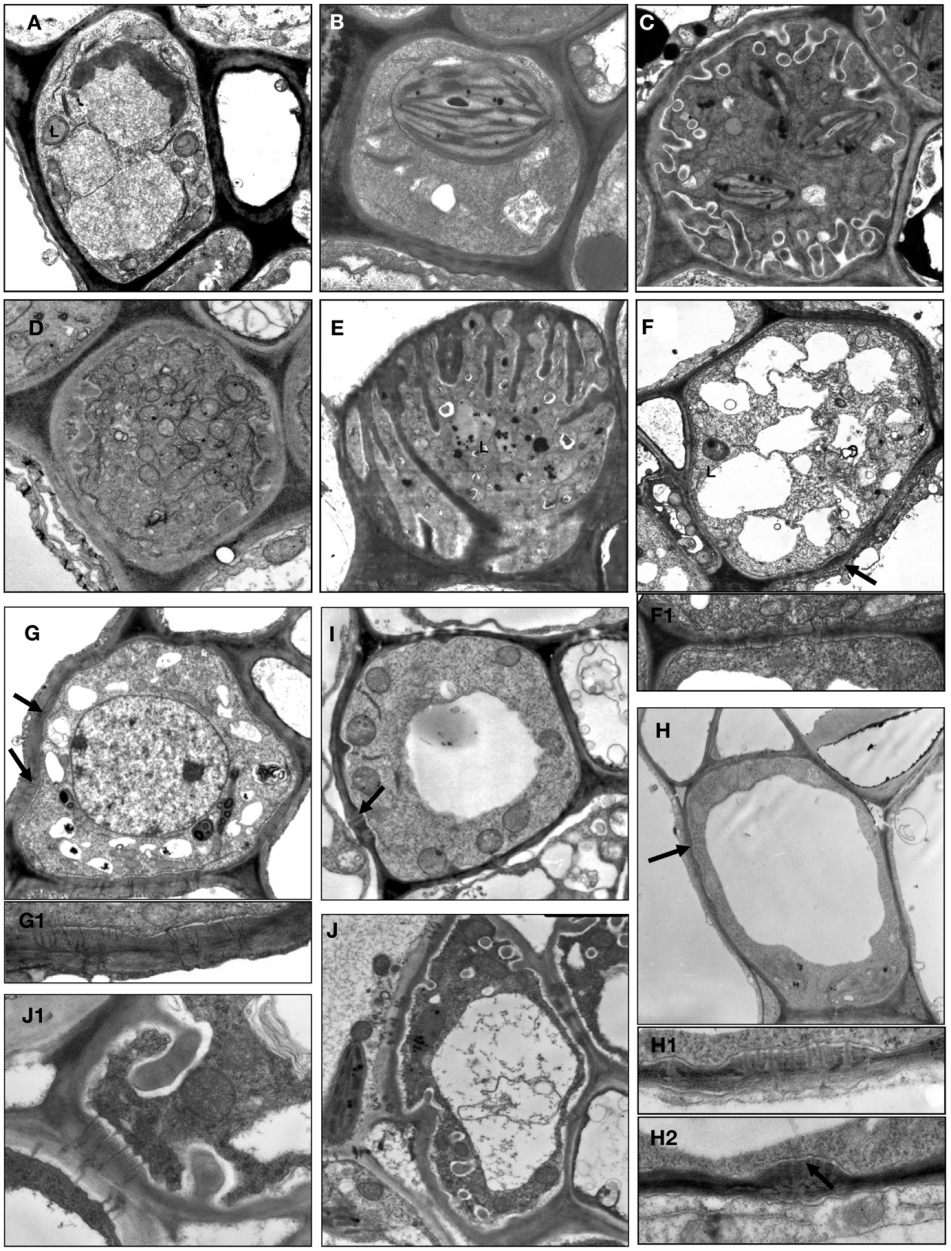
**Structural diversity of companion cells in minor veins in representative species according to classification in Table 1.** L, leucoplast; arrow points on plasmodesmal fields between companion cells and bundle sheath cells at several subfigures. **(A)** *Swertia obtusa*, “ordinary cell” OC-a: plasmodesmal fields and protuberances of the cell wall are absent; plastids are leucoplasts. **(B)** *Vinca minor*, “ordinary cell” OC-b: neither protuberances of the cell wall nor plasmodesmal fields are present; plastids are well-developed chloroplasts. **(C)** *Onosma gmelinii*, “transfer cell” TC-a: multiple protuberances of the cell wall are present while no plasmodesmal fields are formed; plastids are well-developed chloroplasts. **(D)** *Dracocephalum peregrinum*, “transfer cell” TC-b with leucoplasts (not shown) in the abaxial sieve element-companion cell complex (two other sieve element-companion cell complexes in this minor vein contain intermediary cells). **(E)** *Gentiana aquatica*, “transfer cell” TC-c: multiple protuberances of the cell wall of unusual flange morphology are present, plasmodesmal fields are absent, plastids are leucoplasts. **(F)** *Hamelia patens*, companion cell presumably (as no data on RFO synthesis are available) of “intermediary cell” type with large plasmodemal fields, leucoplasts and a number of fragmented vacuoles; the detail in **F1** shows a plasmodesmal field with branched plasmodesmata, ×15,000. **(G)** *Catesbaea spinosa*, companion cell ICL with large plasmodemal fields; plastids are leucoplasts containing starch; the detail in **G1** below shows a plasmodesmal field with branched plasmodesmata, ×15,000. **(H)** *Amsonia tabernaemontana*, companion cell CC-b with well-developed plasmodesmal fields (the details in **H1** and **H2** below, ×15,000) and well-developed chloroplasts; no cell wall protuberances are formed, and one (shown) or a few (not shown) relatively large vacuoles are present. **(I)** *Asarina barclaiana* “modified intermediary cells” MIC-a containing small plasmodesmal fields and small cell wall ingrowths. **(J)** *Rhinanthus minor*, companion cell MIC-b with well-developed plasmodesmal fields, cell wall ingrowths, and leucoplasts; detail in **J1** shows fragment of the cell containing both cell wall protuberances and a plasmodesmal field with magnification ×10,000. Magnification: ×8000 if not otherwise indicated.

On the basis of the features listed above, eleven varieties of companion cell were distinguished in Asteridae (Table 1; Figure 1); however, some of them were widespread and others rare (family- or species-specific; see below). These structural varieties were ranked according to subtypes and grouped into four major companion cell types: OCs, TCs, ICs and IC-like cells, and CC with plasmodesmal fields/many single plasmodesmata including modified intermediary cells (MICs; Turgeon et al., 1993).

**Table 1:** **Classification of companion cells in Asteridae according to their structural characteristics**.

**CC type**	**CC subtype**	**PF**	**Branching of PD in PF**	**CWI**	**Morphology of CWI**	**Chloroplasts (instead of leucoplasts)**	**Stachyose synthesis**
Ordinary cells	OC-a (OC with leucoplasts)^1^	–	–	–	–	–	–
	OC-b (OC with chloroplasts)^2^	–	–	–	–	+	–
Transfer cells	TC-a^3^	–	–	+++	reticulate	+	–
	TC-b^4^ (present in minor veins together with IC/MIC)	–	–	++	reticulate	–	–
	TC-c (in some Gentianaceae species; this study)	–	–	+++	flange	–	–
Intermediary cells and IC-like cells	IC^5^	+++	asymmetric	–	–	– (starch in leucoplasts never observed)	+++
	ICL (IC-like cells with starch in leucoplasts; this study)	+++	asymmetric	–	–	– (starch in leucoplasts normally found)	?
CC with PF and/or many PD	CC-a (CC in minor veins of Cornaceae, Eucommiaceae, Griseliniaceae and Hydrangeaceae; this study)	++	symmetric	–	–	–	–
	CC-b (CC in minor veins of *Amsonia tabernaemontana* and some other Apocynaceae; this study)	++	Asymmetric (no branching in case of multiple single PD)	–	–	+	–
	MIC-a^6^	+	asymmetric	+	reticulate	–	+
	MIC-b (CC in minor veins of some hemiparasitic Orobanchaceae; this study)	+++	asymmetric	+++	reticulate	–	–(?)

CC, companion cell; PF, plasmodesmal fields; PD, plasmodesmata; CWI, cell wall ingrowths. Number of + indicates the relative degree of the manifestation of a feature. Several examples illustrating the trait combinations can be found in

1 Turgeon et al. (1993) for Digitalis grandiflora,

2 Reidel et al. (2009) for Plantago major,

3 Gunning and Pate (1969) for Impatiens balsamina,

4 Turgeon et al. (1993) for Nemesia strumosa,

5 Fisher (1986) for Coleus blumei;

6 *Turgeon et al. (1993) for Asarina scandens; −, feature absent; ?, not clear*.

OCs, sometimes referred to simply as “companion cells,” which are characterized by the absence of both plasmodesmal fields and cell wall ingrowths, contained two subtypes with either leucoplasts (OC-a; Figure 1A) or chloroplasts (OC-b; Figure 1B), respectively (Table 1). TCs were classified on the basis of plastid type and morphology of cell wall ingrowths. The companion cells of TC type described by Pate and Gunning as “A-type transfer cells” contained chloroplasts (Pate and Gunning, 1969); a cell of that type (TC-a in the present classification) is shown in Figure 1C. Companion cells of TC type containing solely leucoplasts were observed in phloem endings in two cases: either in combination with ICs or MICs (e.g., Turgeon et al., 1993. for *Nemesia strumosa*), or, as the only type of phloem companion cells, in minor veins of some representatives of the Gentianaceae family (Batashev and Gamalei, 2000). Figure 1D illustrates a TC found in minor vein phloem together with ICs; these TCs possess leucoplasts and small cell-wall protuberances and were named TC-b. Figure 1E shows TCs which were found only in some representatives of the Gentianaceae family; these TCs possess cell wall ingrowths with flange morphology rather than reticulate morphology as typically observed in companion cells (Offler et al., 2003). This feature was consistently observed in four out of 15 studied Gentianaceae species and was found to be independent of the angle of sectioning. Companion cells of this structure, to our knowledge, have not been reported before in any other species. We named these cells TC-c (Table 1).

Companion cells with abundant plasmodesmal fields in the cell walls facing the bundle sheath and with leucoplasts are widespread in the Asteridae. First, there are ICs, a type of companion cells with very distinct features. The main structural characteristic of IC is the presence of highly developed plasmodesmal fields, asymmetrically branched, with more branches on the IC side (Gamalei, 1974; Turgeon et al., 1975; Volk et al., 1996). To date, a perfect correlation exists between the presence of the ICs and the synthesis of RFOs as phloem transport sugars. Another well-known structural trait of ICs is the presence of many small vacuoles instead of one large vacuole; these unusual vacuoles were found in all ICs of stachyose-transporting plants leading to the speculation that they might represent the compartment for RFO synthesis (Gamalei, 1990; Voitsekhovskaja, 2002). However, we did not include this feature in the present classification because, in contrast to other features, the presence and the number of these vacuoles strongly depend on the physiological condition of the plant (Gamalei et al., 2000). In the present study, we classified the companion cells only on the basis of their stable structural features. An example of a companion cell, the structure of which is similar to that of ICs but for which no analyses of transport sugars have been performed is shown in Figure 1F for *Hamelia patens* (Rubiaceae). This cell has asymmetrical branching of plasmodesmata in the fields facing the bundle sheath (Figure 1F, detail 1), and possesses leucoplasts.

In many species, companion cells with abundant plasmodesmal fields in the cell walls facing the bundle sheath could be found which resembled ICs in some respects but differed in others. We refer to these cells here as ICL (intermediary-cells-like). These cells contained multiple plasmodesmal fields with asymmetrical branching of plasmodesmata and leucoplasts but their leucoplasts contained starch while leucoplasts in ICs had never been shown to accumulate starch. To our knowledge, this cell type has not been described before. One example of IC-like cells with starch-containing leucoplasts is shown in Figure 1G for *Catesbaea spinosa* (Rubiaceae); for this species, no phloem sap analysis has been performed. Interestingly, small vacuoles were present in most cases in such cells. Such companion cells with starch-containing leucoplasts are obviously different from ICs and referred to as ICL in Table 1.

Companion cells with multiple plasmodesmal fields, or sometimes many single plasmodesmata (see below) were also found in Asteridae. In representatives of Cornaceae and Hydrangeaceae (Cornales), Griseliniaceae (Apiales, former Cornales) and Eucommiaceae (Garryales), companion cells possessed leucoplasts without starch and in most cases small vacuoles; however, multiple plasmodesmal fields contained only symmetrically branched plasmodesmata (i.e., with similar number of branches on both sides). Companion cells of closely similar structure have been described, e.g., for poplar (Russin and Evert, 1985) or for *Liquidambar styraciflua* by Turgeon and Medville (2004); these authors found that they were functionally quite different from ICs in that they were not involved in loading RFOs in the phloem. These cells are referred to as CC-a in Table 1. Furthermore, companion cells with abundant plasmodesmal fields which could not be classified as ICs were found in several Apocynaceae species. These companion cells contained chloroplasts; the plasmodesmal fields were found in local thickenings of the cell walls facing the bundle sheath which contained both branched (with more branches at the CC side) and simple plasmodesmata, in contrast to the situation in ICs where only highly branched plasmodesmata with more branches at the IC side were observed in plasmodesmal fields (Figure 1H). In four of these species, *Allamanda cathartica, Alstonia macrophylla, Plumeria rubra*, and *Thevetia nereifolia*, leaf sugars were analyzed by GC-MS but no members of the RFO family were detected (data not shown) which, taking into account the high sensitivity of the method, means that these cells do not synthesize RFO. Such companion cells, to our knowledge, have not been described previously. We refer to these cells as CC-b (Table 1).

Quite peculiar companion cells which, to our knowledge, have not been described before were found in minor vein phloem of several hemiparasitic Orobanchaceae species of the genera *Euphrasia, Melampyrum, Odontites*, and *Rhinanthus*. These cells contained plasmodesmal fields with numerous asymmetrically branched plasmodesmata, resembling those in ICs, along with highly developed cell wall ingrowths (Figure 1J). The plastids found in such cells were leucoplasts which in some cases contained one or two single thylakoids; the presence of chloroplasts could not be confirmed. Combinations of plasmodesmal fields and cell wall protuberances were previously reported for MICs described for *Asarina scandens* (Turgeon et al., 1993) and *A. barclaiana* (Voitsekhovskaja et al., 2006; Figure 1I). However, contrary to the companion cells of the hemiparasitic Orobanchaceae, MIC in *Asarina* species contained only small plasmodesmal fields and only few cell wall ingrowths. Sugar analyses in leaves of *Euphrasia, Melampyrum*, and *Rhinanthus* species revealed high amounts of sucrose and a sugar alcohol galactitol (dulcitol) but no raffinose or stachyose (data not shown). We classified these companion cells as MIC-a for *Asarina* species, and MIC-b for hemiparasitic Orobanchaceae, respectively (Table 1).

### Spatial organization of minor veins in asteridae and classification of minor vein phloem into subtypes

**Figure 2:**
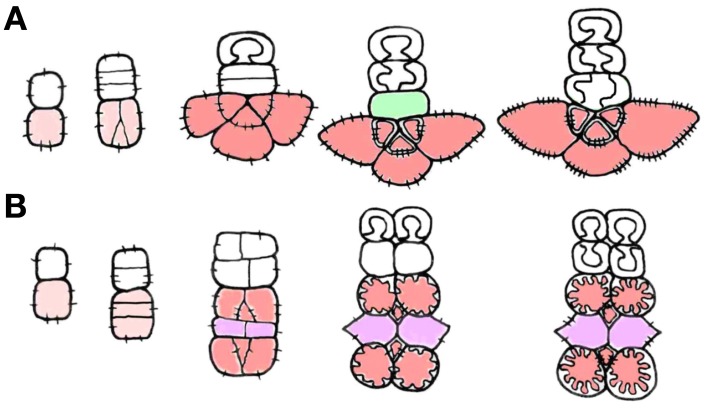
**Schemes showing cell division planes in course of minor vein development [redrawn from Gamalei (1990)]. (A)** type 1; **(B)** type 2. Phloem initial cell and mother cell of the phloem complex are shown in pink, SE-CCCs are shown in red, and phloem parenchyma cells are shown in purple. The vascular parenchyma is shown in green.

The analysis of companion cell types in Asteridae raised the questions as to how various subtypes of companion cells can be combined in a phloem ending, and how the newly described structures are related to the spatial organization of minor veins. In type 1 (“open” type) minor veins as described by Gamalei (1989) for *Syringa vulgaris*, the first divisions of the phloem initial are anticlinal, sometimes occurring as an anticlinal bifurcation. Therefore, one initial produces two to three cells at once. Each of them subsequently undergoes one unequal division. The smaller cell differentiates in a sieve element, and the larger cell in a companion cell. As a result, two or three SE-CCCs form an arc around the abaxial pole of the radial xylem ray in a mature minor vein of type 1 (Figure 2A). This sequence of cell divisions resembles the radial divisions characteristic for the cambium of trees. The xylem and phloem parts of the minor vein in type 1 are usually separated by a row of parenchyma cells designated either as vascular parenchyma, or as phloem parenchyma, which is not quite correct because these cells do not originate from the phloem initial. Here these cells are referred to as vascular parenchyma. In type 2 (“closed” type) minor veins, the first divisions of xylem and phloem initials in course of minor vein formation are periclinal; only the last divisions in the group of phloem cells are anticlinal (original micrographs shown in Gamalei, 1989, for *Senecio sp*.). As a result, the phloem cells form several layers (“tiers”) which are situated one under another (Figure 2B). These tiers consist either of SE-CCCs or of phloem parenchyma cells. Here, xylem and phloem are not separated by a layer of parenchyma cells.

In the present study, the layout of minor veins in most studied species corresponded to either type 1 or 2 (Supplemental Table 1). The following traits were attributed to type 1: (i) minor veins show a well-defined spatial organization; (ii) the veins can contain companion cells of more than one structural type; (iii) the number of companion cells equals the number of sieve elements (CC:SE = 1) which results from the pattern of cell divisions during minor vein formation; (iv) phloem initials do not form phloem parenchyma cells (but vascular parenchyma is present, see above) in the course of minor vein development. The minor veins of type 2 showed the following traits: (i) minor veins show a well-defined spatial organization, (ii) companion cells within the vein belong to a single structural type; (iii) the CC:SE ratio equals 2 or 1.5 (for instance when two SE-CCCs are combined in one minor vein, one with the ratio of CC:SE = 1 and the second with a ratio of CC:SE = 2); (iv) phloem parenchyma is present, and phloem parenchyma and SE-CCCs are positioned in alternating layers.

Apart from these types, a small group of species with minor veins of variable composition resulting from unordered anticlinal and periclinal divisions during vein formation was found in the Asteridae. In this review, we refer to this group as type 0; it consists mostly of representatives of the 1-2a group according to Gamalei (1989, 1991). In this type, (i) the spatial organization of minor veins is not well-defined; (ii) the number of CCs exceeds the number of SEs; (iii) the CC:SE ratio is not stable; (iv) phloem parenchyma cells can be present or absent. Interestingly, companion cells in type 0 minor veins studied thus far were never found to contain highly specialized structures like plasmodesmal fields or a cell wall labyrinth. A published example of this minor vein organization is *Digitalis grandiflora* (Turgeon et al., 1993).

In types 1 and 2, several subtypes can be distinguished on the basis of (i) presence of plasmodesmal fields at companion cell/bundle sheath boundary, (ii) type of plastids in companion cells, (iii) presence of cell wall ingrowths in companion cells, (iv) presence and position of phloem parenchyma cells within minor veins, and (v) presence of cell wall ingrowths in phloem parenchyma cells (Table 2). The presence or absence of these characteristics can be clearly seen on almost any transversal section. This categorization does not include subordinate features such as type of plasmodesmata branching, type of transported sugars or presence of starch in leucoplasts of IC-like cells. The full information on the structural features of the minor vein phloem in the 320 analyzed species is shown in the Supplemental Table 1.

**Table 2:** **Classification of the minor vein phloem according to its spatial organization in leaves of Asteridae**.

**Type**	**Subtype**	**Vein symmetry**	**CC subtype**	**Number of CC subtypes combined in one phloem ending**	**Phloem parenchyma**	**CWI in phloem parenchyma**	**Examples**
1	1-I	+	IC, ICL, CC-a	1	−	−	Figure 4A
	1-II	+	IC,OC-a	2	−	−	Figure 4B
	1-III	+	IC or MIC-a	2	−	−	Figure 4C
		(rarely −)	TC-b				
	1-IV	+	CC-b	1	−	−	Figure 4D
0	0	−	OC-a, OC-b	1	− (rarely +)	−	Figure 4F
2	2-I	+	OC-a, OC-b	1	+	−	Figure 5A
	2-II	+	OC-a, OC-b	1	+	+	Figure 5B
		(rarely −)					
	2-III	+	TC-a	1	+	−	Figure 5C
	2-IV	+	TC-a	1	+	+	Figure 5D
	2-V	+	TC-c	1	+	−	Figure 5E
	2-VI	+/−	MIC-b	1	+	−	Figure 5F

CWI, cell wall ingrowths; CC, companion cells. “+” marks the presence and “−” the absence of a feature.

**Figure 3:**
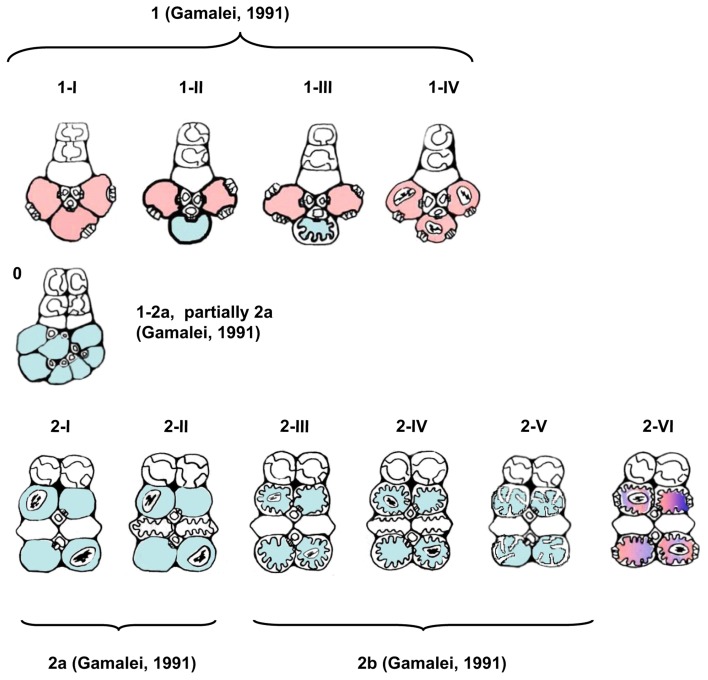
**Schematic presentation of the structural types and subtypes of minor vein phloem.** Absence of plasmodesmal fields at the companion cell/bundle sheath interface is designated by blue, and the presence—by red color; in the types where companion cells could never be shown to contain chloroplasts, plastids are not depicted. Classification into four types according to Gamalei (1991) is shown for reference.

**Figure 4:**
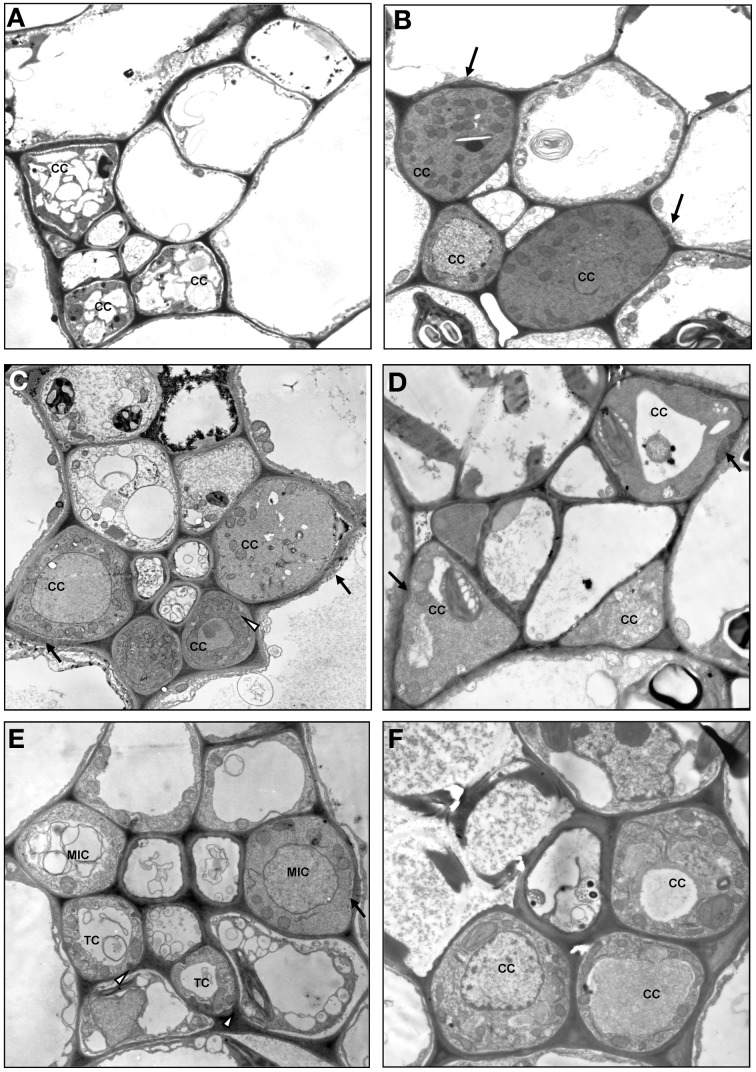
**Structure of minor vein phloem, type 1, and type 0. (A)** *Scutellaria pallida*, subtype 1-I; **(B)** *Mimulus guttatus*, subtype 1-II; **(C)** *Dracocephalum peregrinum*, subtype 1-III; **(D)** *Amsonia tabernaemontana*, subtype 1-IV; **(E)** *Asarina barclaiana*; **(F)** *Trachelospermum luikinense*, type 0. CC, companion cell; MIC-a, modified intermediary cell subtype a; SE, sieve element; TC, transfer cell subtype b. Black arrows point on plasmodesmal fields, white arrowheads point on cell wall ingrowths. Magnification: **(A–D), (F)**, ×1750; **(E)**, ×3000.

Type 1 was divided into subtypes I-IV (Figures 3, 4A–D). In the subtype 1-I, the minor veins contain three SE-CCCs with companion cells of a similar structure (IC, ICL, or CC-a) (Table 1; Figure 4A). This is one of the most widespread subtypes in the Asteridae occurring in the Acanthaceae, Apocynaceae, Bignoniaceae, Cornaceae, Eucommiaceae, Griseliniaceae, Gesneriaceae, Hydrangeaceae, Lamiaceae, Oleaceae, Orobanchaceae, Pawlowniaceae, Plantaginaceae, Rubiaceae, Scrophulariaceae, and Verbenaceae. Subtype 1-II is very close to 1-I; the only difference is that in minor veins with three SE-CCCs, the abaxial SE-CCC contains an OC-a, a companion cell without plasmodesmal fields and cell wall ingrowths (Figure 4B). The subtype 1-II is also quite widespread and was found in Acanthaceae, Bignoniaceae, Lamiaceae, Orobanchaceae, Phrymaceae, Plantaginaceae, Rubiaceae, Scrophulariaceae, and Verbenaceae. Sometimes, both subtypes 1-I and 1-II occurred simultaneously in leaves of the same plant. In species classified subtype 1-III, in minor veins with three SE-CCCs the abaxial SE-CCC contains a companion cell of type TC-b (Table 1), i.e., a TC with leucoplasts (Figure 4C). This subtype was found in Acanthaceae, Lamiaceae, Plantaginaceae, and Scrophulariaceae.

In subtype 1-IV the SE-CCCs contain CC-b companion cells with plasmodesmal fields and chloroplasts (Table 1; Figure 4D). In this subtype, all companion cells within the minor vein are of similar structure. This subtype was found in several genera of the Apocynaceae family, e.g., *Alstonia, Alyxia, Amsonia* (Supplemental Table 1; Batashev and Gamalei, 2005). A similar organization of minor veins was found in several representatives of Campanulaceae and Convolvulaceae, with the exception that the companion cells contained no distinct plasmodesmal fields but rather highly abundant single plasmodesmata (Madore et al., 1986; Supplemental Table 1). Whether phloem loading in these families is similar to, or distinct from that in the representatives of the Apocynaceae with minor vein subtype 1-IV, needs further investigation.

**Figure 5:**
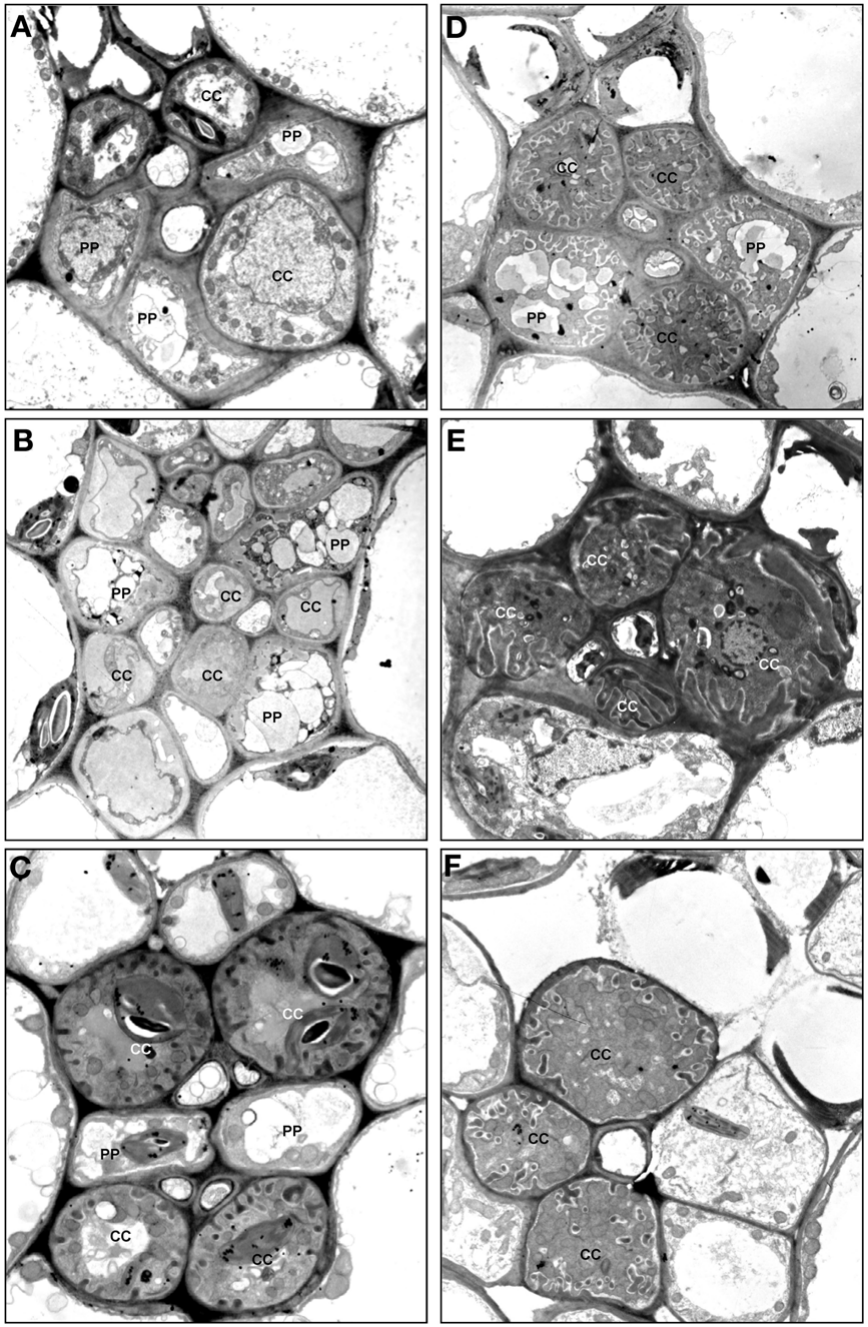
**Structure of minor vein phloem, type 2. (A)** *Ehretia cordifolia*, subtype 2-I; **(B)** *Veronica chamaedrys*, subtype 2-II; **(C)** *Galium krylovianum*, subtype 2-III; **(D)** *Aster alpinus*, subtype 2-IV; **(E)** *Gentiana aquatica*, subtype 2-V; **(F)** *Rhinanthus minor*, subtype 2-VI. CC, companion cell; PP, phloem parenchyma; SE, sieve element. Magnification: **(A,F)** ×1700; **(B)** ×2000; **(C, E)** ×3000; **(D)** ×4000.

Type 2 was divided into subtypes I-VI (Figures 3, 5A–F). Subtype 2-I comprises species with SE-CCCs containing OC-b cells with chloroplasts (in rare cases OC-a with leucoplasts instead of chloroplasts) and phloem parenchyma cells without cell wall ingrowths (Figure 5A). This subtype was found in some representative of the Gentianaceae, Plantaginaceae, and Polemoniaceae, but was broadly represented in the families Boraginaceae and Solanaceae. Subtype 2-II is distinct from the previous subtype in that the phloem parenchyma cells in the minor veins contains cell wall ingrowths and thus can be classified as phloem parenchyma transfer cells (Figure 5B). This subtype occurs very rarely in Asteridae and was found only in Polemoniaceae and Plantaginaceae. Subtype 2-III is widespread and can be found in Asteraceae, Boraginaceae, Plantaginaceae, Rubiaceae, and Solanaceae. It is characterized by the presence of companion cells of TC type with chloroplasts (TC-a, Table 1) while phloem parenchyma cells do not possess cell wall ingrowths (Figure 5C). Subtype 2-IV is very similar to 2-III, the only difference is that in 2-IV cell wall ingrowths are formed in both companion cells and phloem parenchyma cells (Figure 5D). This subtype is common in Asteraceae but rather rare in Solanaceae. Subtype 2-V has been found only in some genera of the Gentianaceae (Supplemental Table 1; Batashev and Gamalei, 2000). The minor veins in these species contain SE-CCCs with rather peculiar transfer cells: their cell wall ingrowths have a flange morphology (Offler et al., 2003), and the cells contain leucoplasts and not chloroplasts (Figure 5E).

Subtype 2-VI has been found in some hemiparasitic representatives of the Orobanchaceae family, *Euphrasia, Melampyrum, Odontites*, and *Rhinanthus*, where SE-CCCs contained MIC-b companion cells with both highly developed cell wall ingrowths and plasmodesmal fields (Figure 5F) and leucoplasts. The mature minor veins of these group of species often showed no well-defined spatial organization, and more detailed studies on the development of the minor veins in these species are required to clarify the relationship of subtype 2-VI with the other types of minor vein phloem. Similarly, the minor vein phloem of the two *Asarina* species, *A. barclaiana* and *A. scandens*, was difficult to include in the subtypes described above, primarily due to lack of data on their minor vein development. In mature minor veins, the abaxial SE-CCCs usually contained two companion cells of TC type per one SE while the adaxial part of the phloem consisted of SE-CCCs with MIC-a, with a MIC:SE ratio of 1 or 2. The absence of phloem parenchyma and of tiered organization, as well as the presence of two different companion cell types within a vein, places these species in type 1, close to subtype 1-III (Figure 4E). At the same time, the companion cells (MICs) in *Asarina* formally resemble those in hemiparasitic Scrophulariaceae, differing from the companion cells of subtype 2-VI in that their plasmodesmal fields and cell wall ingrowths were much less developed. Obviously, the classification of the minor vein phloem in these species needs further investigation.

**Figure 6:**
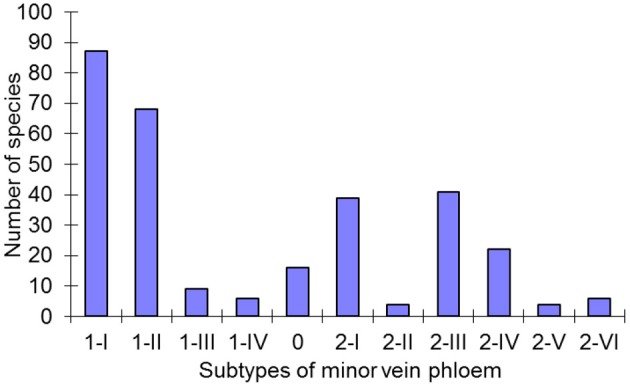
**Distribution of the subtypes of minor vein phloem among 320 species in the Asteridae.** For every subtype defined, the number of species assigned to this subtype is shown.

Type 0 represents a rather heterogeneous group and the rare occurrence of this type in the Asteridae (Figure 6) prevented its comprehensive analysis. The following combinations of structural features could be found within this group: minor veins with SE-CCCs containing OCs with leucoplasts, lacking phloem parenchyma cells, e.g., *Swertia* in Gentianaceae, *Cinchona* in Rubiaceae (Batashev and Gamalei, 2000; Gamalei et al., 2008); minor veins with similar SE-CCCs but containing phloem parenchyma cells (in *Digitalis* species, not shown); similar minor veins where phloem parenchyma cells developed cell wall ingrowths (in several *Veronica* species, not shown); minor veins with SE-CCCs containing OCs with chloroplasts, lacking phloem parenchyma cells (e.g., *Trachelospermum* in Apocynaceae; Figure 4F). The variability of the cellular composition, the absence of highly specialized structures like plasmodesmal fields or cell wall ingrowths, and the lack of well-defined spatial organization distinguish the minor vein phloem of this group from types 1 and 2.

### Distribution of the types and subtypes of minor vein phloem within asteridae

The large number of species analyzed in this study has allowed the determination of the distribution of the defined structural types of minor vein phloem within the studied families of subclass Asteridae (Figure 6). Of the eleven subtypes, five (1-I, 1-II, 2-I, 2-III, and 2-IV) dominate, i.e., were found in over 80% of all species analyzed. The other six subtypes are less wide-spread. The distribution of the types and subtypes of minor vein phloem among the studied families is shown in Table 3. The most heterogeneous family was found to be the Plantaginaceae s.l., where almost all subtypes could be found (Table 3).

**Table 3:** **Distribution of minor vein phloem types and subtypes among the families of Asteridae**.

**Family**	**Subtype of the minor vein phloem**										
	**1-I**	**1-II**	**1-III**	**1-IV**	**0**	**2-I**	**2-II**	**2-III**	**2-IV**	**2-V**	**2-VI**
Acanthaceae	+	+	+								
Apocynaceae	+			+	+	+					
Asteraceae								+	+		
Bignoniaceae	+	+									
Boraginaceae						+		+			
Campanulaceae				+							
Convolvulaceae				+							
Cornaceae	+										
Eucommiaceae	+										
Gentianaceae					+	+				+	
Gesneriaceae	+										
Griseliniaceae	+										
Hydrangeaceae	+										
Lamiaceae	+	+	+								
Oleaceae	+										
Orobanchaceae	+	+									+
Pawlowniaceae	+										
Phrymaceae		+									
Plantaginaceae (Veronicaceae)	+	+	+		+	+	+	+	+		
Polemoniaceae						+	+				
Rubiaceae	+	+			+			+			
Scrophulariaceae	+	+	+								
Solanaceae						+		+	+		
Verbenaceae	+	+									

## Discussion

In this study, we analyzed the minor vein anatomy of 320 species from the subclass Asteridae. First, we performed a cytological analysis of companion cells, and described altogether eleven differentiation forms some of which had not been reported previously. These structures were classified as subtypes within four major types of companion cells; this classification can be extended by adding newly found structural types of companion cells as the cytological studies of companion cells in angiosperms proceed. Furthermore, we investigated the anatomical features of minor vein phloem and developed a classification of minor vein structure in the Asteridae. These two classifications can be used independently, as each has its own range of applications.

We performed separate classifications for companion cell cytology and for minor vein anatomy, respectively, for several reasons. Obviously, it is the cytology of companion cells that defines the potential for symplasmic or apoplasmic loading and thus is relevant for the functional classification of plants with regard to their phloem loading mode. At the same time, many species have more than one type of companion cells in minor veins, and more than one mode of phloem loading, but nevertheless are still referred to as “apoplasmic loaders” and “symplasmic loaders,” following the typology of Gamalei (1991) where the possibility for different types of companion cells to be combined in one phloem ending was not taken into account. To solve this problem, a more detailed classification is to be introduced where attention is paid to all types of companion cells present in one phloem ending.

However, it would be wrong to reduce the classification of minor vein phloem just to a description of the entire assembly of companion cells as this would neglect the evolutionary aspects of minor vein phloem. As shown in this study, plasmodesmal connections or cell wall ingrowths, respectively, can develop in companion cells of minor veins with different ontogenesis. This was not obvious in early studies because the minor vein types 1 and 2 were originally discovered in species among which anatomical, developmental and cytological features were correlated, a fact later referred to as “symplasmic/apoplasmic syndrome” (Gamalei, 2007). The main features, the patterns of cells divisions that give rise to the minor veins, and the numbers of plasmodesmata connecting companion cells and bundle sheath cells showed a very good correlation in that species with ICs (“typical symplasmic loaders”) were always characterized by first anticlinal divisions of the phloem initial and those with TCs (“typical apoplasmic loaders”) by periclinal divisions (Gamalei, 1989). The reasons why in an overwhelming number of species two major types of vein spatial organization correlate with symplasmic continuity remain unclear; however, as shown in this study, there are a few exceptions to this correlation. Thus, the two classifications carry important information, the classification of companion cell ultrastructure for functional studies, and the classification of minor vein structure for developmental and evolutionary studies.

In the first part of our study, we used several structural features to describe the observed diversity of companion cells. The development of cell wall ingrowths, the presence of plasmodesmal fields and the type of plastids in companion cells were independent features, while the form of plasmodemata branching and the morphology of cell wall ingrowths were subordinate features. These traits were correlated in some cases, but not in others. For instance, leucoplasts were always correlated with the presence of asymmetrically branched plasmodesmal fields (and with the synthesis of RFOs) in ICs, and chloroplasts were always correlated with the development of cell wall ingrowths of reticulate morphology in TCs in type 2 minor veins. Generally, chloroplasts occurred in those companion cells types where no plasmodesmal fields developed; the only remarkable exception were ICL-c cells in Apocynaceae species, where chloroplasts were present together with asymmetrically branched plasmodesmal fields. Altogether these cellular structures represented diagnostic features which helped to distinguish between various companion cell types, while their functional significance requires further investigation.

We report here a few novel structures of companion cells. In some representatives of Gentianaceae, TCs showed flange morphology of their cell wall ingrowths, which had been never observed to date in TCs of the phloem origin. Also, new structural types of companion cells were found containing highly developed plasmodesmal fields. Among them, the most interesting type seems to be MIC-b found in some hemiparasitic Orobanchaceae species. Representatives of genera like *Rhinanthus* and *Melampyrum* (subtype 2-VI) provide an interesting model for functional studies because their companion cells possess both well-developed cell wall ingrowths and plasmodesmal fields with no features of plasmodesmal occlusions (Figure 1J, detail 1). The very unusual structure of the companion cells might be related to the hemiparasitism of these species. The plants represent root hemiparasites which gain mineral nutrients and water from their host species. Recent studies showed that they also can receive up to 50% of their carbon budget from the hosts as xylem-mobile organic compounds (Těšitel et al., 2010). This could explain a need for increased apoplasmic uptake of carbon compounds from the xylem into their minor vein phloem for further translocation, along with symplasmic loading of mesophyll sugars derived from photosynthesis as has been shown for monocots (Botha et al., 2008). However, the correlation between hemiparasitism and minor vein phloem subtype disappeared when more species were included in the analysis (Table 4).

**Table 4:** **Type of minor vein phloem (Supplemental Table 1) and hemiparasitism in Orobanchaceae (Gamalei, 2007)**.

**Hemi-parasitic genera**	**Life form**	**Minor vein phloem**
*Bartsia*	Perennial herb	1-I
*Castilleja*	Annual herb	1-II
*Pedicularis*	Perennial herb	1-II
*Euphrasia*	Annual herb	2-VI
*Melampyrum*	Annual herb	2-VI
*Odontites*	Annual herb	2-VI
*Rhinanthus*	Annual herb	2-VI

Interestingly, MIC-b displayed not only plasmodesmal fields and cell wall ingrowths at the interface with the bundle sheath, but they also developed cell wall ingrowths at the walls which directly contacted the xylem. The immediate contact of companion cells and even of sieve elements with xylem elements in minor veins of these species might be a reason for a need to increase the surface of apoplasmic contact between xylem and phloem. It would be interesting to study minor vein structures in these species in course of phloem development and in relation with establishment of hemiparasitism, and to find out which metabolites are present in xylem- and phloem sap, respectively.

The important question of the exact mechanisms of symplasmic phloem loading can be investigated further using several new models described here, such as CC-b in Apocynaceae, MIC-b in hemiparasitic Orobanchaceae, and ICL which seemingly differ from ICs only by the ability to accumulate starch in leucoplasts. In all these cases, there are asymmetrically branched plasmodesmata the exclusion limit of which is probably regulated, analogous to the situation in ICs. The polymer trap model proposing RFO synthesis as a mechanism regulating barrier properties of such plasmodesmata in species with ICs was cast into doubt based on results obtained using the ICs-containing species *Alonsoa meridionalis* (Voitsekhovskaja et al., 2006, 2009); moreover, in leaves of species containing MIC-b and CC-b, no RFOs could be detected by GC-MS which, considering the sensitivity of the method, leads to the conclusion that RFOs cannot be the main transport form in these species, at least not at all developmental stages.

In the analysis of the spatial organization of the minor veins and its relation to the types of companion cells and phloem parenchyma within the veins we summarized the complexes of structural features characteristic of types 1 and 2, and introduced type 0 for minor veins showing unstable structural characteristics. The spatial organization of minor vein phloem results from the patterns of the divisions of phloem initials in course of the minor vein development, and thus has an important evolutionary meaning; this is supported by the fact that minor veins of types 1 and 2 show strong correlations with the growth forms of trees and herbs, respectively (Gamalei, 1989, 1991). The present study showed that the layout of minor vein phloem in an overwhelming number of species correlates with the extent of the development of symplasmic connections between bundle sheath cells and phloem companion cells, confirming earlier conclusions (Gamalei, 1989, 1991). However, several exceptions to this rule were found. First, the hemiparasitic Orobanchaceae species are able to form plasmodesmal fields along with cell wall protuberances in their companion cells, and the spatial organization of the minor veins differed from that in type 1 species. Second, in species of the Campanulaceae and the Convolvulaceae families, the minor vein layout was similar to that of type 1 veins but the veins contained no ICs or IC-like companion cells, and no plasmodesmal fields; their companion cells were connected with the bundle sheath by multiple single plasmodesmata. A detailed study of this minor vein type was published by Madore et al. (1986). These examples show that the two features, the anticlinal position of the first division plane of the phloem initial and the ability to develop highly abundant plasmodesmal fields, although coupled in the majority of species studied thus far, can develop independently. Interestingly, the position of the Campanulaceae on the phylogenetic tree of Asterales is at the base, where shrubs and small trees occur (Lundberg, 2009), but the Asteraceae family at the top of this tree is represented by herbs with minor veins of type 2.

The classification of type 0 is far from complete, since the subclass Asteridae predominantly comprises species with highly specialized minor veins of types 1 or 2 (Figure 6). Type 0 probably could be better resolved via a similar study of the subclass Rosidae which is rich in “type 0 species” (Yu. V. Gamalei, M. V. Pakhomova, and D. R. Batashev, unpublished observations). The new nomenclature presented in this study has been developed for the Asteridae and thus may not be sufficient to cover all dicots; however, it can be easily broadened by adding new subtypes in the course of future studies on the Asteridae or other groups of dicots. Generally, type 1-2a in Gamalei's classification (Gamalei, 1991) is divided between subtypes 0 and 2-I in the nomenclature of this study, while Gamalei's types 1 and 2 keep their positions, but the numbers of subtypes increase (Figure 3). Again, we stress the fact that the 1, 1-2a, 2a, 2b nomenclature is still valuable for characterization of the general ability for the sym- or apoplasmic loading mode. Several years ago, a classification was proposed which might be regarded as consensus between functional and structural descriptions. This classification, which was independently proposed by Gamalei (2000) and van Bel and Hafke (2005), is a combination of ontogenetic and structural views of minor vein phloem. van Bel and Hafke (2005) integrated the functional aspect by including the transport carbohydrates. It includes five subtypes of the minor vein phloem, 0, 1A, 1B, 2A, and 2B, where 0, 1, and 2 refer to minor vein spatial organization and the phloem-loading mode, and A or B refer to less or more advanced structural specialization, respectively.

The data also make clear that in many species with “open” minor vein cytology, i.e., putative symplasmic phloem loaders with multiple plasmodesmata at the bundle sheath/phloem boundary, minor veins often contain a SE-CCC specialized for apoplasmic loading. A well-known example is the minor vein phloem in *Coleus blumei* (Fisher, 1986). The present study provides a quantitative estimate of the occurrence of species of the Asteridae with more than one type of SE-CCC in their minor veins (Figure 6). About half of the “putative symplasmic phloem loaders” in the Asteridae (subtypes 1-II and 1-III) seem to rely on apoplasmic phloem loading in addition to the symplasmic pathway. This proportion might be even higher regarding the fact that also “pure symplasmic loaders” of subtype 1-I can additionally contain minor veins of the 1-II type in their leaves (e.g., *Paederia scandens* or *Scrophularia americana*; Supplemental Table 1). The idea that always both symplasmic and apoplasmic mechanisms contribute to phloem loading, one usually being the dominant loading mode, is not new (see e.g., Kursanov, 1984). However, functional evidence for this idea has been provided only recently using a stachyose-translocating species, *Alonsoa meridionalis*. In minor veins of *A. meridionalis* which belong to the subtype 1-II according to the classification of this study, expression of the stachyose synthase gene *AmSTS1* was found only in the two adaxial SE-CCCs containing ICs while the sucrose transporter *Am*SUT1 was localized at the plasma membrane of the OC in the abaxial SE-CCC (Voitsekhovskaja et al., 2009). These data indicate that apoplasmic loading operates even in species with “open” minor vein anatomy, at least in a large part of them. Yet ca. 30% of the Asteridae examined in this study (97 out of 315 species belonging to subtypes 1-I and 1-IV) seem to use no additional structures specialized for the apoplasmic phloem loading. Interestingly, in the IC-containing species *Cucumis melo*, a switch from symplasmic to apoplasmic loading has been demonstrated upon Cucumber Mosaic Virus infection; moreover, sucrose uptake activity from the apoplast into minor veins was detected in healthy *C. melo* plants (Gil et al., 2011). Further studies are required to understand how symplasmic phloem loading works in plants belonging to subtypes 1-I and 1-IV, whether apoplasmic loading takes place, and which mechanisms are used in companion cells of type 1-IV to prevent sucrose leakage into the mesophyll via plasmodesmata in the absence of RFO synthesis.

In conclusion, the detailed analysis of the minor vein phloem in the large group of species from the subclass Asteridae presented in this study shows that there is more variation in minor vein organization than previously known. This opens up the field to further developmental, phylogenetic, and functional analyses of phloem loading. Several points which deserve further investigation are: what are transport sugars and loading mechanisms in subtypes 1-I and 1-IV species (is apoplastic loading involved or not)? What are the mechanisms underlying the strong correlation of type 1/2 minor vein symmetry and the presence/absence of plasmodesmal fields in companion cells? What are phylogenetic relationships between different minor vein types and subtypes? What are the functions of plasmodesmal fields and cell wall ingrowths, respectively, in hemiparasitic Orobanchaceae with MIC-b cells?

### Conflict of interest statement

The authors declare that the research was conducted in the absence of any commercial or financial relationships that could be construed as a potential conflict of interest.
