# Supplementary material for: Cytology of the minor-vein phloem in 320 species from the subclass Asteridae suggests a high diversity of phloem-loading modes
Source: Front Plant Sci. 2013 Aug 21;4:312. doi: 10.3389/fpls.2013.00312 (PMC3748319; doi:10.3389/fpls.2013.00312)
Supplement: Supplementary file 1 [file DataSheet1.DOC]

**Supplement Table 1.** List of species studied for minor vein anatomy, and their structural characteristics. “+” mark the presence and “-” the absence of a characteristic; in case when two CC types are present in the vein, data for each CC are separated by comma. Literature data were from:1 – van Bel et al., 1992; 2 – data from this study and/or unpublished data from Yu.V. Gamalei’s laboratory; 3 – Batashev and Gamalei, 2005; 4 – Pate and Gunning, 1969; 5 – Peterson and Jeung, 1975; 6 – Fisher, 1991; 7 – Evert, 1980; 8 – Madore et al., 1986; 9 – Batashev and Gamalei, 2000; 10 - Madore and Grodzinski, 1984; 11 – Reidel et al., 2009; 12 – Gamalei et al., 2008; 13– Voitsekhovskaja et al., 2006; 14 – Turgeon et al., 1993; 15 – Roberts et al., 1997; 16 – Ding et al., 1988; 17 – McCauley, Evert, 1989; 18 – dos Santos et al., 2011. Data on transported sugars are from Zimmermann and Ziegler, 1975 or references 1-14, as indicated above; data on leaf sugars are from this study or from 18. Abbreviations: Ch, chloroplast; Gal, galactitol; Gol, galactinol; Ino, inositol; L, leucoplasts; M, mannitol; n.d., not determined; R, raffinose; red – reduced; s.pd., single plasmodesmata; St, stachyose; Suc, sucrose; V, verbascose. The sequence of sugars reflects their abundance in the phloem sap; trace amounts are shown in parentheses. Perennial = perennial herb; annual =annual herb; decid. tree = deciduous tree; evergr. tree = evergreen tree. (?), classification needs further clarification. Families are given according to AGPIII (2009).

| Species | | Type of minor vein phloem | Subtype | | Number of CC types | | PF in CC | CWI in CC | | | | | | | Plastid in CC | | Phloem parenchyma present | | | | Sugars transported | | | | Life form | | | Source | | | | |  | | | | | | | | |
| --- | --- | --- | --- | --- | --- | --- | --- | --- | --- | --- | --- | --- | --- | --- | --- | --- | --- | --- | --- | --- | --- | --- | --- | --- | --- | --- | --- | --- | --- | --- | --- | --- | --- | --- | --- | --- | --- | --- | --- | --- | --- |
| Acanthaceae | | | | | | | | | | | | | | | | | | | | | | | | | | | | | | | | |  | | | | | | | | |
| *Acanthus mollis* L. | | 1 | 1-III | | | 2 | +,– | –,+ | | | | | | | L | | – | | | n.d. | | | | perennial | | | | | 1 | | | |  | | | | | | | | |
| *Acanthus spinosus* L. | | 1 | 1-III | | | 2 | +,– | –,+ | | | | | | | L | | – | | | n.d. | | | | perennial | | | | | 2 | | | |  | | | | | | | | |
| *Avicennia tomentosa* Jacq. | | 1 | 1-I | | 1 | | + | – | | | | | | | L | | – | | | n.d. | | | | tree | | | | 2 | | | |  | | | | | | | | | |
| *Asystasia gangetica* (L.) T.Anders | | 1 | 1-II | | | 2 | +,– | – | | | | | | | L | | – | | | n.d. | | | | perennial | | | | | 2 | | | |  | | | | | | | | |
| *Barleria micans* Nees | | 1 | 1-II | | | 2 | +,– | – | | | | | | | L | | – | | | n.d. | | | | tree | | | | | 2 | | | |  | | | | | | | | |
| *Diciptera suberecta* (Andre) Bremekamp | | 1 | 1-III | | | 2 | +,– | –,+ | | | | | | | L | | – | | | n.d. | | | | semishrub | | | | | 2 | | | |  | | | | | | | | |
| *Justicia carnea* Lindl | | 1 | 1-I | | | 1 | + | – | | | | | | | L | | – | | | n.d. | | | | shrub | | | | | 2 | | | |  | | | | | | | | |
| *Justicia gendarussa* L. | | 1 | 1-II | | | 2 | +,– | – | | | | | | | L | | – | | | n.d. | | | | semishrub | | | | | 2 | | | |  | | | | | | | | |
| *Ruellia blumei* Steud. | | 1 | 1-I | | | 1 | + | – | | | | | | | L | | – | | | n.d. | | | | perennial | | | | | 2 | | | |  | | | | | | | | |
| *Thunbergia elegans* Borzi. | | 1 | 1-I | | | 1 | + | – | | | | | | | L | | – | | | n.d. | | | | shrub | | | | | 2 | | | |  | | | | | | | | |
| Apocynaceae | | | | | | | | | | | | | | | | | | | | | | | | | | | | | | | | |  | | | | | | | | |
| *Acokanthera oppositifolia* (Lam.) Codd | | 1 | 1-I | | 1 | | + | – | | | | | | | L | | | – | | n.d. | | | | tree | | | | | | 3 | | |  | | | | | | | | |
| *Allamanda cathartica* L. | | 1 | 1-IV | | 1 | | + | – | | | | | | | Ch | | | – | | Suc*** | | | | tree | | | | | | 2, 3 | | |  | | | | | | | | |
| *Alstonia macrophylla* Wall. et G.Don | | 1 | 1-IV | | 1 | | + | – | | | | | | | Ch | | | – | | Suc*** | | | | tree | | | | | | 2, 3 | | |  | | | | | | | | |
| *Alyxia daphnoides* A.Cunn. | | 1 | 1-IV | | 1 | | + | – | | | | | | | Ch | | | – | | n.d. | | | | tree | | | | | | 3 | | |  | | | | | | | | |
| *Amsonia tabernaemontana* Walt. | | 1 | 1-IV | | 1 | | + | – | | | | | | | Ch | | | – | | n.d. | | | | tree | | | | | | 3 | | |  | | | | | | | | |
| *Araujia sericifera* Brot. | | 0 | 0 | | 1 | | – | – | | | | | | | Ch | | + | | | n.d. | | | | semishrub | | | | | | 2 | | |  | | | | | | | | |
| *Asclepias curassavica* L. | | 0 | 0 | | 2 | | – | – | | | | | | | L,Ch | | + | | | n.d. | | | | semishrub | | | | | | 2 | | |  | | | | | | | | |
| *Carissa carandas* L. | | 1 | 1-IV | | 1 | | + | – | | | | | | | Ch | | | – | | n.d. | | | | tree | | | | | | 3 | | |  | | | | | | | | |
| *Cynanchum sibiricum* Willd. | | 0 | 0 | | 1 | | – | – | | | | | | | L | | + | | | n.d. | | | | perennial | | | | | | 2 | | | | | |  | | | | | |
| *Kopsia fruticosa* (Ker.) A.DC. | | 1 | 1-I | | 1 | | + | – | | | | | | | L | | | – | | Suc, R, St | | | | tree | | | | | | 3 | | |  | | | | | | | | |
| *Nerium oleander* L. | | 1 | 1-I | | 1 | | + | – | | | | | | | L | | | – | | Suc, R, St, V | | | | tree | | | | | | 3 | | |  | | | | | | | | |
| *Pachypodium lamerei* Drake | | 1 | 1-I | | 1 | | + | – | | | | | | | L | | | – | | n.d. | | | | tree | | | | | | 3 | | |  | | | | | | | | |
| *Plumeria rubra* L. | | 1 | 1-IV | | 1 | | + | – | | | | | | | L | | | – | | Suc*** | | | | tree | | | | | | 2, 3 | | |  | | | | | | | | |
| *Rauvolfia perakensis* King et Gahble | | 1 | 1-I | | 1 | | + | – | | | | | | | L | | | – | | n.d. | | | | tree | | | | | | 3 | | |  | | | | | | | | |
| *Stapelia grandiflora* Masson* | | 0 | 0 | | 1 | | – | – | | | | | | | L | | + | | | n.d. | | | | succulent | | | | | | 2 | | | | | |  | | | | | |
| *Thevetia nereifolia* Juss. ex Steud. | | 1 | 1-IV | | 1 | | + | – | | | | | | | Ch | | | – | | Suc*** | | | | tree | | | | | | 2, 3 | | |  | | | | | | | | |
| *Trachelospermum asiaticum* Nakai. | | 0 | 0 | | 1 | | – | – | | | | | | | Ch | | | – | | n.d. | | | | shrub | | | | | | 3 | | |  | | | | | | | | |
| *Trachelospermum jasminoides* (Lindl.) Lem. | | 0 | 0 | | 1 | | – | – | | | | | | | Ch | | | – | | n.d. | | | | shrub | | | | | | 3 | | |  | | | | | | | | |
| *Trachelospermum lukinense* Hatus. | | 0 | 0 | | 1 | | – | – | | | | | | | Ch | | | – | | n.d. | | | | shrub | | | | | | 3 | | |  | | | | | | | | |
| *Vinca minor* L. | | 0 | 0 | | 1 | | – | – | | | | | | | Ch | | | + | | n.d. | | | | perennial | | | | | | 3 | | |  | | | | | | | | |
| *Vinca major* L. | | 0 | 0 | | 1 | | – | – | | | | | | | Ch | | | + | | n.d. | | | | perennial | | | | | | 3 | | |  | | | | | | | | |
| *Vincetoxicum sibiricum* (L.) Decne | | 0 | 0 | | 1 | | – | – | | | | | | | L,Ch | | + | | | n.d. | | | | perennial | | | | | | 2 | | | | | |  | | | | | |
| *Voacanga papuana* Schumann | | 1 | 1-I | | 1 | | + | – | | | | | | | L | | | – | | n.d. | | | | tree | | | | | | 3 | | |  | | | | | | | | |
| *Wrightia religiosa* ( Teysm. et Binn.) Benyh. | | 1 | 1-I | | 1 | | + | – | | | | | | | L  (starch) | | | - | | n.d. | | | | tree | | | | | | 3 | | |  | | | | | | | | |
| Asteraceae | | | | | | | | | | | | | | | | | | | | | | | | | | | | | | | | | | | |  | | | | | |
| *Achillea apiculata* Orlova | | 2 | 2-IV | | 1 | | – | + | | | | | | | Ch | | + | | | n.d. | | | | perennial | | | | | 2 | | | | | | |  | | | | | |
| *Achillea millefolium* L. | | 2 | 2-IV | | 1 | | – | + | | | | | | | Ch | | + | | | n.d. | | | | perennial | | | | | 2 | | | | | | |  | | | | | |
| *Anacyclus pyrethrum* DC. | | 2 | 2-IV | | 1 | | – | + | | | | | | | Ch | | + | | | n.d. | | | | perennial | | | | | 4 | | | | | | |  | | | | | |
| *Antennaria dioica* (L.) Gaertn. | | 2 | 2-IV | | 1 | | – | + | | | | | | | Ch | | + | | | n.d. | | | | perennial | | | | | 2 | | | | | | |  | | | | | |
| *Artemisia caespitosa* Ledeb. | | 2 | 2-III | | 1 | | – | + | | | | | | | Ch | | + | | | n.d. | | | | perennial | | | | | 2 | | | | | | |  | | | | | |
| *Artemisia frigida* Willd. | | 2 | 2-III | | 1 | | – | + | | | | | | | Ch | | + | | | n.d. | | | | perennial | | | | | 2 | | | | | | |  | | | | | |
| *Artemisia mongolica* (Bess.) Fisch.ex Nakai | | 2 | 2-III | | 1 | | – | + | | | | | | | Ch | | + | | | n.d. | | | | perennial | | | | | 2 | | | | | | |  | | | | | |
| *Artemisia scoparia* Waldst & Kit. | | 2 | 2-III | | 1 | | – | + | | | | | | | Ch | | + | | | n.d. | | | | perennial | | | | | 2 | | | | | | |  | | | | | |
| *Cacalia hastata* L. | | 2 | 2-III | | 1 | | – | + | | | | | | | Ch | | + | | | n.d. | | | | perennial | | | | | 2 | | | | | | |  | | | | | |
| *Chrysanthemum sp.* | | 2 | 2-IV | | 1 | | – | + | | | | | | | Ch | | + | | | n.d. | | | | annual | | | | | 2 | | | | | | |  | | | | | |
| *Eupatorium atrorubens* | | 2 | 2-IV | | 1 | | – | + | | | | | | | Ch | | + | | | n.d. | | | | tree | | | | | 2 | | | | | | |  | | | | | |
| *Eupatorium lindleyanum* DC. | | 2 | 2-III | | 1 | | – | + | | | | | | | Ch | | + | | | n.d. | | | | perennial | | | | | 2 | | | | | | |  | | | | | |
| *Filago arvensis* L. | | 2 | 2-III | | 1 | | – | + | | | | | | | Ch | | + | | | n.d. | | | | annual | | | | | 2 | | | | | | |  | | | | | |
| *Flaveria brownii* | | 2 | 2-III | | 1 | | – | + | | | | | | | Ch | | + | | | n.d. | | | | perennial | | | | | 2 | | | | | | |  | | | | | |
| *Helianthus annuus* L. | | 2 | 2-IV | | 1 | | – | + | | | | | | | Ch | | + | | | n.d. | | | | annual | | | | | 2 | | | | | | |  | | | | | |
| *Hieracium florbundum* Wimm & Grab. | | 2 | 2-IV | | 1 | | – | + | | | | | | | Ch | | + | | | n.d. | | | | perennial | | | | | 5 | | | | | | |  | | | | | |
| *Inula helenium* L. | | 2 | 2-III | | 1 | | – | + | | | | | | | Ch | | + | | | n.d. | | | | perennial | | | | | 5 | | | | | | |  | | | | | |
| *Lactuca sibirica* (L.) Maxim. | | 2 | 2-III | | 1 | | – | + | | | | | | | Ch | | + | | | n.d. | | | | perennial | | | | | 5 | | | | | | |  | | | | | |
| *Leontopodium leontopodioides* (Willd.) Beauverd | | 2 | 2-IV | | 1 | | – | + | | | | | | | Ch | | + | | | n.d. | | | | perennial | | | | | 5 | | | | | | |  | | | | | |
| *Ligularia altaica* D.C. | | 2 | 2-III | | 1 | | – | + | | | | | | | Ch | | + | | | n.d. | | | | perennial | | | | | 5 | | | | | | |  | | | | | |
| *Ligularia kaepferi* Siebold et Zuci | | 2 | 2-IV | | 1 | | – | + | | | | | | | Ch, L, | | + | | | n.d. | | | | perennial | | | | | 5 | | | | | | |  | | | | | |
| *Olearia odorata* Petri | | 2 | 2-IV | | 1 | | – | + | | | | | | | Ch | | + | | | n.d. | | | | shrub | | | | | 5 | | | | | | |  | | | | | |
| *Pulicaria vulgaris* Gaertn. | | 2 | 2-III | | 1 | | – | + | | | | | | | Ch | | + | | | n.d. | | | | annual | | | | | 4 | | | | | | |  | | | | | |
| *Rudbeckia laciniata* L. | | 2 | 2-III | | 1 | | – | + | | | | | | | Ch | | + | | | n.d. | | | | annual | | | | | 2 | | | | | | |  | | | | | |
| *Saussurea amara* L.DC. | | 2 | 2-IV | | 1 | | – | + | | | | | | | Ch | | + | | | n.d. | | | | perennial | | | | | 2 | | | | | | |  | | | | | |
| *Sausssurea involucrata* (Kar.& Kir.) Sch. Bip. | | 2 | 2-IV | | 1 | | – | + | | | | | | | Ch | | + | | | n.d. | | | | perennial | | | | | 2 | | | | | | |  | | | | | |
| *Saussurea schanginiana* (Wydi.) Fsch. ex Herd. | | 2 | 2-IV | | 1 | | – | + | | | | | | | Ch | | + | | | n.d. | | | | perennial | | | | | 2 | | | | | | |  | | | | | |
| *Senecio anteuphorbium* (L.) Sch. Bip. | | 2 | 2-IV | | 1 | | – | + | | | | | | | Ch | | + | | | n.d. | | | | succulent | | | | | 2 | | | | | | |  | | | | | |
| *Senecio grandifolius* Less. | | 2 | 2-IV | | 1 | | – | + | | | | | | | Ch | | + | | | n.d. | | | | tree | | | | | 2 | | | | | | |  | | | | | |
| *Senecio rowleyanus* Jacobs. | | 2 | 2-IV | | 1 | | – | + | | | | | | | Ch | | + | | | n.d. | | | | succulent | | | | | 2 | | | | | | |  | | | | | |
| *Senecio vernalis* W.K. | | 2 | 2-IV | | 1 | | – | + | | | | | | | Ch | | + | | | Suc | | | | annual | | | | | 2 | | | | | | |  | | | | | |
| *Senecio vulgaris* L. | | 2 | 2-III | | 1 | | – | + | | | | | | | Ch | | + | | | Suc | | | | annual | | | | | 4 | | | | | | |  | | | | | |
| *Solidago virgaurea* L. | | 2 | 2-IV | | 1 | | – | + | | | | | | | Ch | | + | | | n.d. | | | | perennial | | | | | 2 | | | | | | |  | | | | | |
| *Sonchus oleraceus* L. | | 2 | 2-III | | 1 | | – | + | | | | | | | Ch | | + | | | Suc | | | | annual | | | | | 6 | | | | | | |  | | | | | |
| *Tagetes patula* L. | | 2 | 2-III | | 1 | | – | + | | | | | | | Ch | | + | | | n.d. | | | | annual | | | | | 7 | | | | | | |  | | | | | |
| *Xanthium strumarium* L. | | 2 | 2-IV | | 1 | | – | + | | | | | | | Ch | | + | | | n.d. | | | | annual | | | | | 2 | | | | | | |  | | | | | |
| Bignoniaceae | | | | | | | | | | | | | | | | | | | | | | | | | | | | | | | | | | | |  | | | | | |
| *Campsis fragrans* | | 1 | 1-II | | 2 | | + | – | | | | | | | L | | – | | | n.d. | | | | decid. tree | | | | | 2 | | | | | | |  | | | | | |
| *Campsis radicans* Seem. | | 1 | 1-II | | 2 | | + | – | | | | | | | L | | – | | | St, Suc, R, Ino | | | | decid. tree | | | | | 2 | | | | | | |  | | | | | |
| *Catalpa bignonioides* Walt. | | 1 | 1-I(1-II) | | 1(2) | | +(+,-) | – | | | | | | | L | | – | | | Suc, St, R, V | | | | decid. tree | | | | | 2 | | | | | | |  | | | | | |
| *Catalpa longissima* (Jacq.) Sims | | 1 | 1-I(1-II) | | 1(2) | | +(+,-) | – | | | | | | | L | | – | | | St, R, Suc | | | | tree | | | | | 2 | | | | | | |  | | | | | |
| *Jacaranda sograeana* D.C. | | 1 | 1-I | | 1 | | + | – | | | | | | | L | | – | | | St, R, Suc | | | | evergr. tree | | | | | 2 | | | | | | |  | | | | | |
| *Kigelia pinnata* (Jacq.) D.C. | | 1 | 1-I | | 1 | | + | – | | | | | | | L | | – | | | n.d. | | | | evergr. tree | | | | | 2 | | | | | | |  | | | | | |
| *Spathodea campanulata* P.Beauv. | | 1 | 1-I | | 1 | | + | – | | | | | | | L | | – | | | St, R, Suc | | | | evergr. tree | | | | | 2 | | | | | | |  | | | | | |
| *Spathodea nilotica* Seem. | | 1 | 1-I | | 1 | | + | – | | | | | | | L | | – | | | St, R (Suc) | | | | evergr. tree | | | | | 2 | | | | | | |  | | | | | |
| *Stereospermum kunthianum* Cham. | | 1 | 1-I | | 1 | | + | – | | | | | | | L | | – | | | St, R (Suc) | | | | evergr. tree | | | | | 2 | | | | | | |  | | | | | |
| *Tabebuia berteri* Britton | | 1 | 1-I | | 1 | | + | – | | | | | | | L | | – | | | St, Suc, R | | | | evergr. tree | | | | | 2 | | | | | | |  | | | | | |
| *Tabebuia glomerata* Urban | | 1 | 1-I | | 1 | | + | – | | | | | | | L | | – | | | St, Suc (R, V) | | | | evergr. tree | | | | | 2 | | | | | | |  | | | | | |
| *Tabebuia heterophylla* Britt. | | 1 | 1-I | | 1 | | + | – | | | | | | | L | | – | | | St, Suc, R | | | | evergr. tree | | | | | 2 | | | | | | |  | | | | | |
| *Tabebuia leucoxyla* P.DC | | 1 | 1-I | | 1 | | + | – | | | | | | | L | | – | | | St, R, Suc | | | | evergr. tree | | | | | 2 | | | | | | |  | | | | | |
| *Tabebuia pallida* Miers. | | 1 | 1-I | | 1 | | + | – | | | | | | | L | | – | | | St, Suc, R (V) | | | | evergr. tree | | | | | 2 | | | | | | |  | | | | | |
| *Tabebuia speciosa* Standley | | 1 | 1-I | | 1 | | + | – | | | | | | | L | | – | | | St, Suc, R | | | | evergr. tree | | | | | 2 | | | | | | |  | | | | | |
| Boraginaceae | | | | | | | | | | | | | | | | | | | | | | | | | | | | | | | | | | | |  | | | | | |
| *Cordia angiocarpa* A. Rich. | | 2 | 2-I | 1 | | | – | – | | | | | | | Ch | | – | | | Suc | | | | tree | | | | | 2 | | | | | | |  | | | | | |
| *Cordia dichothoma* Forst. | | 2 | 2-I | 1 | | | – | – | | | | | | | Ch | | – | | | Suc | | | | tree | | | | | 2 | | | | | | |  | | | | | |
| *Cordia francisci* Tenore | | 2 | 2-I | 1 | | | – | – | | | | | | | Ch | | + | | | n.d. | | | | shrub | | | | | 2 | | | | | | |  | | | | | |
| *Cordia glabra* Cham. | | 2 | 2-I | 1 | | | – | – | | | | | | | Ch | | – | | | Suc | | | | tree | | | | | 2 | | | | | | |  | | | | | |
| *Cordia myxa* L. | | 2 | 2-I | 1 | | | – | – | | | | | | | Ch | | – | | | Suc | | | | tree | | | | | 2 | | | | | | |  | | | | | |
| *Cordia nitida* Vahl. | | 2 | 2-I | | 1 | | – | – | | | | | | | Ch | | + | | | Suc | | | | perennial | | | | | 2 | | | | | | |  | | | | | |
| *Corida obliqua* Willd. | | 2 | 2-I | | 1 | | – | – | | | | | | | Ch | | + | | | Suc | | | | perennial | | | | | 2 | | | | | | |  | | | | | |
| *Cordia superba* Cham. | | 2 | 2-I | | 1 | | – | – | | | | | | | Ch | | + | | | Suc | | | | perennial | | | | | 2 | | | | | | |  | | | | | |
| *Cordia tremula* Griseb. | | 2 | 2-I | | 1 | | – | – | | | | | | | Ch | | + | | | Suc | | | | perennial | | | | | 2 | | | | | | |  | | | | | |
| *Ehretia cordifolia* C.H. Wright | | 2 | 2-I | | 1 | | – | – | | | | | | | Ch | | + | | | n.d. | | | | shrub | | | | | 2 | | | | | | |  | | | | | |
| *Ehretia thyrsifolia* Nakai | | 2 | 2-I | | 1 | | – | – | | | | | | | Ch | | + | | | Suc (R) | | | | tree. | | | | | 2 | | | | | | |  | | | | | |
| *Eritrichium villosum.* (Ledeb.) Bunge | | 2 | 2-III | | 1 | | – | + | | | | | | | Ch | | + | | | n.d. | | | | perennial | | | | | 2 | | | | | | |  | | | | | |
| *Lithospermum arvense* L. | | 2 | 2-III | | 1 | | – | + | | | | | | | Ch | | + | | | n.d. | | | | annual | | | | | 2 | | | | | | |  | | | | | |
| *Lycopsis arvensis* L. | | 2 | 2-III | | 1 | | – | + | | | | | | | Ch | | + | | | n.d. | | | | annual | | | | | 2 | | | | | | |  | | | | | |
| *Mertensia davurica* (Pali. ex Sims) G.Don fil. | | 2 | 2-III | | 1 | | – | + | | | | | | | Ch | | + | | | n.d. | | | | annual | | | | | 2 | | | | | | |  | | | | | |
| *Myosotis asiatica* (Vesterg.) Schischkin & Sergievskaja | | 2 | 2-III | | 1 | | – | + | | | | | | | Ch | | + | | | n.d. | | | | perennial | | | | | 2 | | | | | | |  | | | | | |
| *Onosma gmelinii* Ledeb. | | 2 | 2-III | | 1 | | – | + | | | | | | | Ch | | + | | | n.d. | | | | perennial | | | | | 2 | | | | | | |  | | | | | |
| *Patogonula americana* L*.* | | 2 | 2-III | | 1 | | – | + | | | | | | | Ch | | + | | | n.d. | | | | tree | | | | | 2 | | | | | | |  | | | | | |
| *Symphytum officinale* L. | | 2 | 2-III | | 1 | | – | + | | | | | | | Ch | | + | | | n.d. | | | | annual | | | | | 2 | | | | | | |  | | | | | |
| *Tournefortia argentea L. fil. = Argusia argentea* | | 2 | 2-I | | 1 | | – | – | | | | | | | Ch | | + | | | n.d. | | | | tree | | | | | 2 | | | | | | |  | | | | | |
| Campanulaceae | | | | | | | | | | | | | | | | | | | | | | | | | | | | | | | | | | | |  | | | | | |
| *Adenophora stenanthina* (Ledeb.) Kitag.** | | 1 | 1-IV (?) | 1 | | | + (rare), s.pd. | – | | | | | | | Ch | | – | | | n.d. | | | | perennial | | | | | 2 | | | | | | |  | | | | | |
| *Adenophora tricuspidata* (Fish. ex Schult..) A.DC.** | | 1 | 1-IV (?) | 1 | | | + (rare), s.pd. | – | | | | | | | Ch | | – | | | n.d. | | | | perennial | | | | | 2 | | | | | | |  | | | | | |
| *Azorina vidalii* (Watson) Feer** | | 1 | 1-IV (?) | 1 | | | + (rare), s.pd. | – | | | | | | | Ch | | – | | | n.d. | | | | perennial | | | | | 2 | | | | | | |  | | | | | |
| *Campanula kemulariae* Fomin** | | 1 | 1-IV (?) | 1 | | | + (rare), s.pd. | – | | | | | | | Ch | | – | | | n.d. | | | | perennial | | | | | 2 | | | | | | |  | | | | | |
| *Campanula patula* L.** | | 1 | 1-IV (?) | 1 | | | + (rare), s.pd. | – | | | | | | | Ch | | – | | | n.d. | | | | perennial | | | | | 2 | | | | | | |  | | | | | |
| *Musschia aurea*** | | 1 | 1-IV (?) | 1 | | | + (rare), s.pd. | – | | | | | | | Ch | | – | | | n.d. | | | | shrub | | | | | 2 | | | | | | |  | | | | | |
| *Trachelium caeruleum*** | | 1 | 1-IV (?) | 1 | | | + (rare), s.pd. | – | | | | | | | Ch | | – | | | n.d. | | | | shrub | | | | | 2 | | | | | | |  | | | | | |
| Convolvulaceae | | | | | | | | | | | | | | | | | | | | | | | | | | | | | | | | | | | |  | | | | | |
| *Calystegia sepium* (L.) R. Br. | | 1 | 1-IV (?) | | 1 | | s.pd | – | | | | | | | Ch | | – | | | n.d. | | | | liana | | | | | | 2 | | | | | |  | | | | | |
| *Convolvulus ammanii* Desr. | | 1 | 1-IV (?) | | 1 | | s.pd. | – | | | | | | | Ch | | – | | | n.d. | | | | liana | | | | | | 2 | | | | | |  | | | | | |
| *Convolvulus arvensis* L. | | 1 | 1-IV (?) | | 1 | | s.pd. | – | | | | | | | Ch | | – | | | n.d. | | | | liana | | | | | | 2 | | | | | |  | | | | | |
| *Ipomoea tricolor* Cav. | | 1 | 1-IV (?) | | 1 | | s.pd. | – | | | | | | | Ch | | – | | | n.d. | | | | liana | | | | | | 8 | | | | | |  | | | | | |
| Cornaceae | | | | | | | | | | | | | | | | | | | | | | | | | | | | | | | | | | | |  | | | | | |
| *Cornus controversa* Hemsl. | | 1 | 1-I | | 1 | | + | - | | | | | | | L | | – | | | n.d. | | | | tree | | | | | | 2 | | | | | |  | | | | | |
| Eucommiaceae | | | | | | | | | | | | | | | | | | | | | | | | | | | | | | | | | | | |  | | | | | |
| *Eucommia ulmoides* Oliv. | | 1 | 1-I | 1 | | | + | - | | | | | | | L | | – | | | n.d. | | | tree | | | | | | | 2 | | | | | |  | | | | | |
| Gentianaceae | | | | | | | | | | | | | | | | | | | | | | | | | | | | | | | | | | | |  | | | | | |
| *Anagalidium dichotomum* (L.) Griseb. | | 2 | 2-I | 1 | | | – | – | | | | | | | L. | | + | | | n.d. | | | annual | | | | | | | 9 | | | | | |  | | | | | |
| *Blackstonia perfoliata* (L.)Hudson | | 2 | 2-I | 1 | | | – | – | | | | | | | L. | | + | | | n.d. | | | annual | | | | | | | 9 | | | | | |  | | | | | |
| *Gentiana algida* Pall. = *Gentianoides algida* | | 2 | 2-V | 1 | | | – | + | | | | | | | L. | | + | | | n.d. | | | perennial | | | | | | | 9 | | | | | |  | | | | | |
| *Gentiana aquatica* L. | | 2 | 2-V | 1 | | | – | + | | | | | | | L. | | + | | | n.d. | | | annual | | | | | | | 9 | | | | | |  | | | | | |
| *Gentiana decumbens* L. fil. | | 2 | 2-I | 1 | | | – | – | | | | | | | L. | | + | | | n.d. | | | perennial | | | | | | | 9 | | | | | |  | | | | | |
| *Gentiana fischeri* P. Smirn. | | 2 | 2-I | 1 | | | – | – | | | | | | | L. | | + | | | n.d. | | | perennial | | | | | | | 9 | | | | | |  | | | | | |
| *Gentiana macrophylla* Pall. | | 2 | 2-I | 1 | | | – | – | | | | | | | L. | | + | | | n.d. | | | perennial | | | | | | | 9 | | | | | |  | | | | | |
| *Gentiana squarrosa* Ledeb. | | 2 | 2-V | 1 | | | – | + | | | | | | | L. | | + | | | n.d. | | | annual | | | | | | | 9 | | | | | |  | | | | | |
| *Gentiana tenella* Rottb. | | 2 | 2-I | 1 | | | – | – | | | | | | | L. | | + | | | n.d. | | | annual | | | | | | | 9 | | | | | |  | | | | | |
| *Gentiana uniflora* Georgi. | | 2 | 2-V | 1 | | | – | + | | | | | | | L. | | + | | | n.d. | | | annual | | | | | | | 9 | | | | | |  | | | | | |
| *Gentianella azurea* Bunge | | 2 | 2-I | 1 | | | – | – | | | | | | | L. | | + | | | n.d. | | | annual | | | | | | | 9 | | | | | |  | | | | | |
| *Gentianopsis ciliata* (L.) Ma | | 2 | 2-I | 1 | | | – | – | | | | | | | L. | | + | | | n.d. | | | perennial | | | | | | | 9 | | | | | |  | | | | | |
| *Halenia corniculata*(L.) Cornaz. | | 2 | 2-I | 1 | | | – | – | | | | | | | L. | | + | | | n.d. | | | annual | | | | | | | 9 | | | | | |  | | | | | |
| *Lomatogonium carrintiacum (*Wulfen) Reichenbach | | 2 | 2-I | 1 | | | – | – | | | | | | | L. | | + | | | n.d. | | | perennial | | | | | | | 9 | | | | | |  | | | | | |
| *Swertia obtusa* Ledeb. | | 0 | 0 | 1 | | | – | – | | | | | | | L. | | – | | | n.d. | | | perennial | | | | | | | 9 | | | | | |  | | | | | |
| Gesneriaceae | | | | | | | | | | | | | | | | | | | | | | | | | | | | | | | | | | | |  | | | | | |
| *Achimenes sp.* | | 1 | 1-I | | 1 | | + | – | | | | | | | L | | – | | | n.d. | | | perennial | | | | | | | 2 | | | | | |  | | | | | |
| *Aeschynanthus boscheanus* De Vrisese | | 1 | 1-I | | 1 | | + | – | | | | | | | L | | – | | | n.d. | | | perennial | | | | | | | 2 | | | | | |  | | | | | |
| *Cyrtandra occidentalis* N.P.Balakr. & B.L.Burtt | | 1 | 1-I | | 1 | | + | – | | | | | | | L | | – | | | n.d. | | | perennial | | | | | | | 2 | | | | | |  | | | | | |
| *Episcia reptans* Mart. | | 1 | 1-I | | 1 | | + | – | | | | | | | L | | – | | | n.d. | | | perennial | | | | | | | 2 | | | | | |  | | | | | |
| *Gloxinia perennis* | | 1 | 1-I | | 1 | | + | – | | | | | | | L | | – | | | n.d. | | | perennial | | | | | | | 2 | | | | | |  | | | | | |
| *Hypocyrta glabra* Hook. | | 1 | 1-I | | 1 | | + | – | | | | | | | L | | – | | | n.d. | | | perennial | | | | | | | 2 | | | | | |  | | | | | |
| *Koleria bogotensis* (Nichols.) Fritsch | | 1 | 1-I | | 1 | | + | – | | | | | | | L | | – | | | n.d. | | | perennial | | | | | | | 2 | | | | | |  | | | | | |
| *Saintpaulia ionantha* | | 1 | 1-I | | 1 | | + | – | | | | | | | L | | – | | | n.d. | | | perennial | | | | | | | 2 | | | | | |  | | | | | |
| *Streptocarpus sp.* | | 1 | 1-I | | 1 | | + | – | | | | | | | L | | – | | | n.d. | | | perennial | | | | | | | 2 | | | | | |  | | | | | |
| Griseliniaceae | | | | | | | | | | | | | | | | | | | | | | | | | | | | | | | | | | | |  | | | | | |
| *Griselinia littoralis* (Raoul.) Raoul. | | 1 | 1-I | 1 | | | + | - | | | | | | | L | | – | | | n.d. | | | tree | | | | | | | 2 | | | | | |  | | | | | |
| Hydrangeaceae | | | | | | | | | | | | | | | | | | | | | | | | | | | | | | | | | | | |  | | | | | |
| *Deutzia scabra* (Raoul.) Raoul. | | 1 | 1-I | 1 | | | + | - | | | | | | | L | | – | | | n.d. | | | shrub | | | | | | | 2 | | | | | |  | | | | | |
| Lamiaceae | | | | | | | | | | | | | | | | | | | | | | | | | | | | | | | | | | | |  | | | | | |
| *Ajuga chia* Schreb. | | 1 | 1-II | | 2 | | +,– | – | | | | | L | | | | – | | | n.d. | | | shrub | | | | | | 2 | | | | | | |  | | | | | |
| *Ajuga reptans* L. | | 1 | 1-II | | 2 | | +,– | – | | | | | L | | | | – | | | n.d. | | | perennial | | | | | | 2 | | | | | | |  | | | | | |
| *Betonica officinalis* L. = *Stachys officinalis* L. | | 1 | 1-II | | 2 | | +,– | – | | | | | L | | | | – | | | n.d. | | | perennial | | | | | | 2 | | | | | | |  | | | | | |
| *Callicarpa arborea* Roxb | | 1 | 1-I | | 1 | | + | – | | | | L | | | | – | | | | | St, Suc, R, V | tree | | | | | | | 2 | | | | |  | | | | | | | |
| *Caryopteris mongolica* Bunge | | 1 | 1-I | | 1 | | + | – | | | | L | | | | – | | | | | n.d. | semishrub | | | | | | | 2 | | | | |  | | | | | | | |
| *Clerodendrum trichotomum* Thunb. | | 1 | 1-I | | 1 | | + | – | | | | L | | | | – | | | | | St, Suc, R, V | tree | | | | | | | 2 | | | | |  | | | | | | | |
| *Dracocephalum geterophyllum* Benth. | | 1 | 1-II | | 2 | | +,– | – | | | | | L | | | | – | | | n.d. | | | perennial | | | | | | 2 | | | | | | |  | | | | | |
| *Dracocephalum peregrinum* L. | | 1 | 1-III | | 2 | | +,– | –,+ | | | | | L | | | | – | | | n.d. | | | perennial | | | | | | 2 | | | | | | |  | | | | | |
| *Dracocephalum thymiflorum* L. | | 1 | 1-II | | 2 | | +,– | – | | | | | L | | | | – | | | n.d. | | | perennial | | | | | | 2 | | | | | | |  | | | | | |
| *Galeopsis speciosa* Mill. | | 1 | 1-II | | 2 | | +,– | – | | | | | L | | | | – | | | n.d. | | | perennial | | | | | | 2 | | | | | | |  | | | | | |
| *Glechoma hederacea* L. | | 1 | 1-II | | 2 | | +,– | – | | | | | L | | | | – | | | n.d. | | | perennial | | | | | | 2 | | | | | | |  | | | | | |
| *Lagopsis marrubiastrum* (Steph.) Ik. - Gal. | | 1 | 1-II | | 2 | | +,– | – | | | | | L | | | | – | | | n.d. | | | perennial | | | | | | 2 | | | | | | |  | | | | | |
| *Lamium album* L. | | 1 | 1-I | | 1 | | + | – | | | | | L | | | | – | | | St | | | perennial | | | | | | 2 | | | | | | |  | | | | | |
| *Lamium purpureum* L. | | 1 | 1-II | | 2 | | +,– | – | | | | | L | | | | – | | | n.d. | | | perennial | | | | | | 2 | | | | | | |  | | | | | |
| *Lavandula spica* L. | | 1 | 1-I | | 1 | | + | – | | | | | L | | | | – | | | n.d. | | | shrub | | | | | | 2 | | | | | | |  | | | | | |
| *Leonurus glaucescen*s Bunge | | 1 | 1-II | | 2 | | +,– | – | | | | | L | | | | – | | | n.d. | | | perennial | | | | | | 2 | | | | | | |  | | | | | |
| *Lophantus chinensis Benth.* | | 1 | 1-III | | 2 | | +,– | –,+ | | | | | L | | | | – | | | n.d. | | | perennial | | | | | | 2 | | | | | | |  | | | | | |
| *Lycopus europaeus L.* | | 1 | 1-I(1-II) | | 1(2) | | +(+,–) | – | | | | | L | | | | – | | | n.d. | | | perennial | | | | | | 2 | | | | | | |  | | | | | |
| *Mentha aquatica L.* | | 1 | 1-II | | 2 | | +,– | – | | | | | L | | | | – | | | n.d. | | | perennial | | | | | | 2 | | | | | | |  | | | | | |
| *Mentha austriaca* | | 1 | 1-II | | 2 | | +,– | – | | | | | L | | | | – | | | n.d. | | | perennial | | | | | | 2 | | | | | | |  | | | | | |
| *Nepeta pannonica*L. | | 1 | 1-I | | 1 | | + | – | | | | | L | | | | – | | | n.d. | | | perennial | | | | | | 2 | | | | | | |  | | | | | |
| *Origanum vulgare* L. | | 1 | 1-I | | 1 | | + | – | | | | | L | | | | – | | | n.d. | | | perennial | | | | | | 2 | | | | | | |  | | | | | |
| *Perilla frutescens (L.) Britt. (P. ocymoides L.)* | | 1 | 1-II | | 2 | | +,– | – | | | | | L | | | | – | | | n.d. | | | perennial | | | | | | 2 | | | | | | |  | | | | | |
| *Phlomis fruticosa* L. | | 1 | 1-I | | 1 | | + | – | | | | | L | | | | – | | | n.d. | | | shrub | | | | | | 2 | | | | | | |  | | | | | |
| *Phlomis tuberosa L.* | | 1 | 1-II | | 2 | | +,– | – | | | | | L | | | | – | | | n.d. | | | perennial | | | | | | 2 | | | | | | |  | | | | | |
| *Prunella laciniata (L.)L.* | | 1 | 1-II | | 2 | | +,– | – | | | | | L | | | | – | | | n.d. | | | perennial | | | | | | 2 | | | | | | |  | | | | | |
| *Prunella vulgaris L.* | | 1 | 1-II | | 2 | | +,– | – | | | | | L | | | | – | | | n.d. | | | perennial | | | | | | 2 | | | | | | |  | | | | | |
| *Rosmarinus officinalis* L. | | 1 | 1-I | | 1 | | + | – | | | | | L | | | | – | | | Suc, R, St | | | shrub | | | | | | 2 | | | | | | |  | | | | | |
| *Salvia aethiopsisL.* | | 1 | 1-II | | 2 | | +,– | – | | | | | L | | | | – | | | n.d. | | | perennial | | | | | | 2 | | | | | | |  | | | | | |
| *Salvia horminum L. = S.viridis* | | 1 | 1-II | | 2 | | +,– | – | | | | | L | | | | – | | | n.d. | | | perennial | | | | | | 2 | | | | | | |  | | | | | |
| *Salvia nemorosa L.* | | 1 | 1-II | | 2 | | +,– | – | | | | | L | | | | – | | | n.d. | | | perennial | | | | | | 2 | | | | | | |  | | | | | |
| *Salvia splendens* Ker-Gawl | | 1 | 1-I | | 1 | | + | – | | | | | L | | | | – | | | n.d. | | | perennial | | | | | | 10 | | | | | | |  | | | | | |
| *Salvia verticillata L.* | | 1 | 1-II | | 2 | | +,– | – | | | | | L | | | | – | | | n.d. | | | perennial | | | | | | 2 | | | | | | |  | | | | | |
| *Salvia viridisL.* | | 1 | 1-II | | 2 | | +,– | – | | | | | L | | | | – | | | n.d. | | | perennial | | | | | | 2 | | | | | | |  | | | | | |
| *Scutellaria albida* L. | | 1 | 1-I | | 1 | | + | – | | | | | L | | | | – | | | n.d. | | | perennial | | | | | | 2 | | | | | | |  | | | | | |
| *Scutellaria baicalensis Georgi* | | 1 | 1-II | | 2 | | +,– | – | | | | | L | | | | – | | | n.d. | | | perennial | | | | | | 2 | | | | | | |  | | | | | |
| *Scutellaria galericulata* L. | | 1 | 1-I | | 1 | | + | – | | | | | L | | | | – | | | n.d. | | | perennial | | | | | | 2 | | | | | | |  | | | | | |
| *Scutellaria karjaginii Grossh.* | | 1 | 1-II | | 2 | | +,– | – | | | | | L | | | | – | | | n.d. | | | perennial | | | | | | 2 | | | | | | |  | | | | | |
| *Scutellaria pallida Bieb. = S.albida* | | 1 | 1-II | | 2 | | +,– | – | | | | | L | | | | – | | | n.d. | | | perennial | | | | | | 2 | | | | | | |  | | | | | |
| *Sideritis comosa (Rochei ex Benth.) Stank.* | | 1 | 1-II | | 2 | | +,– | – | | | | | L | | | | – | | | n.d. | | | perennial | | | | | | 2 | | | | | | |  | | | | | |
| *Stachys iberica* Bieb. | | 1 | 1-I | | 2 | | +,– | – | | | | | L | | | | – | | | n.d. | | | perennial | | | | | | 2 | | | | | | |  | | | | | |
| *Stachys lovandulifolia Vahl* | | 1 | 1-II | | 2 | | +,– | – | | | | | L | | | | – | | | n.d. | | | perennial | | | | | | 2 | | | | | | |  | | | | | |
| *Stachys palustris L.* | | 1 | 1-II | | 2 | | +,– | – | | | | | L | | | | – | | | n.d. | | | perennial | | | | | | 2 | | | | | | |  | | | | | |
| *Tectona grandis* L. | | 1 | 1-II | | 2 | | +,– | – | | | | | | L | | | – | | | St (Suc) | | | tree | | | | | | 2 | | | | | |  | | | | | | |
| *Teucrium chamaedrys L.* | | 1 | 1-II | | 2 | | +,– | – | | | | | L | | | | – | | | n.d. | | | perennial | | | | | | 2 | | | | | | |  | | | | | |
| *Teucrium orientale L.* | | 1 | 1-II | | 2 | | +,– | – | | | | | L | | | | – | | | n.d. | | | perennial | | | | | | 2 | | | | | | |  | | | | | |
| *Thymus serpyllum L.* | | 1 | 1-II | | 2 | | +,– | – | | | | | L | | | | – | | | n.d. | | | perennial | | | | | | 2 | | | | | | |  | | | | | |
| *Thymus subarcticus Klok.* | | 1 | 1-II | | 2 | | +,– | – | | | | | L | | | | – | | | n.d. | | | perennial | | | | | | 2 | | | | | | |  | | | | | |
| *Thymus kotschyanus Bois & Holen.* | | 1 | 1-II | | 2 | | +,– | – | | | | | L | | | | – | | | n.d. | | | perennial | | | | | | 2 | | | | | | |  | | | | | |
| *Vitex agnus-castus* L. | | 1 | 1-I | | 1 | | + | – | L | | | | | | | | – | | Suc, R, St | | | | tree | | | 2 | | | | | | | | | |  | | | | | |
| Oleaceae | | | | | | | | | | | | | | | | | | | | | | | | | | | | | | | | | | | |  | | | | | |
| *Fraxinus americana* L. | | 1 | 1-I | | 1 | | + | – | | | | | L | | | | – | | | St, M,Suc, R (V) | | | tree | | | | 2 | | | | | | | | |  | | | | | |
| *Fraxinus excelsior* L. | | 1 | 1-I | | 1 | | + | – | | | | | L | | | | – | | | St, M,Suc, R (V) | | | tree | | | | 2 | | | | | | | | |  | | | | | |
| *Fraxinus oregona* Nutt. | | 1 | 1-I | | 1 | | + | – | | | | | L | | | | – | | | St, M,Suc, R, V | | | tree | | | | 2 | | | | | | | | |  | | | | | |
| *Fraxinus ornus* L. | | 1 | 1-I | | 1 | | + | – | | | | | L | | | | – | | | St, M,Suc, R, V | | | tree | | | | 2 | | | | | | | | |  | | | | | |
| *Fraxinus oxicarpa* Willd | | 1 | 1-I | | 1 | | + | – | | | | | L | | | | – | | | St, M,Suc, R, V | | | tree | | | | 2 | | | | | | | | |  | | | | | |
| *Jasminium heterophylluum* Рoxb. | | 1 | 1-I | | 1 | | + | – | | | | | L | | | | – | | | M, Suc, St, | | | shrub | | | | 2 | | | | | | | | |  | | | | | |
| *Jasminium nudiflorum* Lindl. | | 1 | 1-I | | 1 | | + | – | | | | | L | | | | – | | | M,Suc, St, R (V) | | | shrub | | | | 2 | | | | | | | | |  | | | | | |
| *Ligustrum japonicum* Thunb. | | 1 | 1-I | | 1 | | + | – | | | | | L | | | | – | | | M,St, Suc, R (V) | | | tree | | | | 2 | | | | | | | | |  | | | | | |
| *Olea europea* L. | | 1 | 1-I | | 1 | | + | – | | | | | L | | | | – | | | M,Suc, St, (R) | | | tree | | | | 2 | | | | | | | | |  | | | | | |
| *Phillyrea decora* Boiss.et Bal. | | 1 | 1-I | | 1 | | + | – | | | | | L | | | | – | | | M,Suc, St (R) | | | shrub | | | | 2 | | | | | | | | |  | | | | | |
| *Phillyrea latifolia* L. | | 1 | 1-I | | 1 | | + | – | | | | | L | | | | – | | | M, Suc, St, R | | | shrub | | | | 2 | | | | | | | | |  | | | | | |
| *Syringa julianae* Сchi | | 1 | 1-I | | 1 | | + | – | | | | | L | | | | – | | | M, St, Suc, R, V | | | tree | | | | 2 | | | | | | | | |  | | | | | |
| *Syringa pubescens* Turcz. | | 1 | 1-I | | 1 | | + | – | | | | | L | | | | – | | | M,St, Suc, R (V) | | | tree | | | | 2 | | | | | | | | |  | | | | | |
| *Syringa vulgaris* L. | | 1 | 1-I | | 1 | | + | – | | | | | L | | | | – | | | St, M, R, V, Suc | | | tree | | | | 2 | | | | | | | | |  | | | | | |
| Orobanchaceae | | | | | | | | | | | | | | | | | | | | | | | | | | | | | | | | | | | |  | | | | | |
| *Bartsia alpina* L. | | 1 | 1-I | | 1 | | + | – | | L | | | | | | | – | | n.d. | | | | perennial | | | 2 | | | | | | | | | |  | | | | | |
| *Castilleja lapponica* Gand. | | 1 | 1-II | | 2 | | +, – | – | | L | | | | | | | – | | n.d. | | | | perennial | | | 2 | | | | | | | | | |  | | | | | |
| *Castilleja pallida (L.) Streng.* | | 1 | 1-II | | 2 | | +, – | – | | L | | | | | | | – | | n.d. | | | | perennial | | | 2 | | | | | | | | | |  | | | | | |
| *Euphrasia fennica Kihlm.* | | 2 | 2-VI | | 1 | | + | + | | L | | | | | | | + | | Suc, Gal, (R,St)*** | | | | annual | | | 2 | | | | | | | | | |  | | | | | |
| *Melampyrum pratense L.* | | 2 | 2-VI | | 1 | | + | + | | L | | | | | | | + | | n.d. | | | | annual | | | 2 | | | | | | | | | |  | | | | | |
| *Melampyrum sylvaticum L.* | | 2 | 2-VI | | 1 | | + | + | | L | | | | | | | + | | Suc, Gal, (R,St)*** | | | | annual | | | 2 | | | | | | | | | |  | | | | | |
| *Odontites vulgaris Moench* | | 2 | 2-VI | | 1-2 | | + | + | | L | | | | | | | + | | n.d. | | | | annual | | | 2 | | | | | | | | | |  | | | | | |
| *Pedicularis abrotanifolia* Bieb. ex Stev. | | 1 | 1-II | | 2 | | +, – | – | | L | | | | | | | – | | St, Suc | | | | perennial | | | 2 | | | | | | | | | |  | | | | | |
| *Pedicularis compacta* Steph. | | 1 | 1-II | | 2 | | +, – | – | | L | | | | | | | – | | St, Suc | | | | perennial | | | 2 | | | | | | | | | |  | | | | | |
| *Pedicularis flava* Pall*.* | | 1 | 1-II | | 2 | | +, – | – | | L | | | | | | | – | | n.d. | | | | perennial | | | 2 | | | | | | | | | |  | | | | | |
| *Pedicularis labradorica* Wirsing | | 1 | 1-II | | 2 | | +, – | – | | L | | | | | | | – | | n.d. | | | | perennial | | | 2 | | | | | | | | | |  | | | | | |
| *Pedicularis lapponica* L. | | 1 | 1-II | | 2 | | +, – | – | | L | | | | | | | – | | n.d. | | | | perennial | | | 2 | | | | | | | | | |  | | | | | |
| *Pedicularis palustris* L. | | 1 | 1-II | | 2 | | +, – | – | | L | | | | | | | – | | n.d. | | | | perennial | | | 2 | | | | | | | | | |  | | | | | |
| *Pedicularis resupinata* L. | | 1 | 1-II | | 2 | | +, – | – | | L | | | | | | | – | | n.d. | | | | perennial | | | 2 | | | | | | | | | |  | | | | | |
| *Pedicularis rubens* Steph*.* | | 1 | 1-II | | 2 | | +, – | – | | L | | | | | | | – | | n.d. | | | | perennial | | | 2 | | | | | | | | | |  | | | | | |
| *Pedicularis sceptrum-carolinum* L*.* | | 1 | 1-II | | 2 | | +, – | – | | L | | | | | | | – | | n.d. | | | | perennial | | | 2 | | | | | | | | | |  | | | | | |
| *Pedicularis spicata* Pall*.* | | 1 | 1-II | | 2 | | +, – | – | | L | | | | | | | – | | n.d. | | | | perennial | | | 2 | | | | | | | | | |  | | | | | |
| *Pedicularis uliginosa* Bunge | | 1 | 1-II | | 2 | | +, – | – | | L | | | | | | | – | | St, Suc | | | | perennial | | | 2 | | | | | | | | | |  | | | | | |
| *Rhinanthus minor* L. | | 2 | 2-VI | | 1 | | + | + | | L | | | | | | | – | | Suc, Gal, (R,St)*** | | | | annual | | | 2 | | | | | | | | | |  | | | | | |
| *Rhinanthus major* L. | | 2 | 2-VI | | 1 | | + | + | | L | | | | | | | – | | n.d. | | | | annual | | | 2 | | | | | | | | | |  | | | | | |
| Pawlowniaceae | | | | | | | | | | | | | | | | | | | | | | | | | | | | | | | | | | | |  | | | | | |
| *Paulownia tomentosa* (Tunb.) Steud. | | 1 | 1-I | | 1 | | + | – | | L | | | | | | | – | | Suc, St, R, V | | | | tree | | | 2 | | | | | | | | | |  | | | | | |
| Phrymaceae | | | | | | | | | | | | | | | | | | | | | | | | | | | | | | | | | | | |  | | | | | |
| *Mimulus cardinalis* Dougl. ex. Benth. | | 1 | 1-II | | 2 | | +, – | – | | L | | | | | | | – | | Suc, R, St, Gol | | | | perennial | | | 14 | | | | | | | | | |  | | | | | |
| *Mimulus guttatus* L. | | 1 | 1-II | | 2 | | +, – | – | | L | | | | | | | – | | Suc, St, R | | | | annual | | | 2 | | | | | | | | | |  | | | | | |
| Plantaginaceae s.l. /Veronicaceae | | | | | | | | | | | | | | | | | | | | | | | | | | | | | | | | | | | |  | | | | | |
| *Antirrihnum majus L.* | | 2 | 2-IV | | 1 | | – | + | | Ch | | | | | | | + | | n.d. | | | | annual | | | 2 | | | | | | | | | |  | | | | | |
| *Asarina scandens (Cav.) Penn.* | | 1 | 1-III(?) | | 2 | | +, – | + | | Ch (red) | | | | | | | + | | Suc, R, St | | | | perennial | | | 14 | | | | | | | | | |  | | | | | |
| *Asarina barclaiana Pennell* | | 1 | 1-III(?) | | 2 | | +, – | + | | L | | | | | | | + | | Suc, R, St, antirrhinoside | | | | perennial | | | 13 | | | | | | | | | |  | | | | | |
| *Cymbalaria muralis Garnet, Mey.& Scherb* | | 2 | 2-III | | 1 | | – | + | | L | | | | | | | + | | Suc, (Gol, R) | | | | perennial | | | 14 | | | | | | | | | |  | | | | | |
| *Digitalis grangiflora* Mill. | | 0 | 0 | | 1 | | – | – | | L | | | | | | | + | | Suc (R, St) | | | | perennial | | | 14 | | | | | | | | | |  | | | | | |
| *Gratiola officinalis* L. | | 0 | 0 | | 1 | | – | – | | L | | | | | | | + | | n.d. | | | | perennial | | | 2 | | | | | | | | | |  | | | | | |
| *Lagotis integripholia* (Wild.) Schischk. | | 1 | 1-II | | 2 | | +, – | – | | L | | | | | | | – | | St, Suc | | | | perennial | | | 2 | | | | | | | | | |  | | | | | |
| *Lagotis uralensis* Schischk*.* | | 1 | 1-II | | 2 | | +, – | – | | L | | | | | | | – | | n.d. | | | | perennial | | | 2 | | | | | | | | | |  | | | | | |
| *Linaria maroccana* Hook.f. | | 2 | 2-III | | 1 | | - | + | | Ch | | | | | | | + | | Suc, (Gol) | | | | annual | | | 14 | | | | | | | | | |  | | | | | |
| *Linaria melampyroides* Kuprian*.* | | 2 | 2-III | | 1 | | - | + | | Ch | | | | | | | + | | n.d. | | | | annual | | | 2 | | | | | | | | | |  | | | | | |
| *Plantago arborescens* Poir. | | 2 | 2-III | | 1 | | – | + | | | Ch | | | | | | + | | n.d. | | | | perennial | | | | 2 | | | | | | | | |  | | | | | |
| *Plantago logopus* L. | | 2 | 2-III | | 1 | | – | + | | | Ch | | | | | | + | | n.d. | | | | perennial | | | | 4 | | | | | | | | |  | | | | | |
| *Plantago major* L. | | 2 | 2-II | | 1 | | – | – | | | Ch | | | | | | + | | n.d. | | | | perennial | | | | 11 | | | | | | | | |  | | | | | |
| *Plantago psillium* L. | | 2 | 2-III | | 1 | | – | + | | | Ch | | | | | | + | | n.d. | | | | perennial | | | | 4 | | | | | | | | |  | | | | | |
| *Plantago spathulata* Hook. | | 2 | 2-I | | 1 | | – | – | | | Ch | | | | | | + | | n.d. | | | | perennial | | | | 4 | | | | | | | | |  | | | | | |
| *Rodochiton atrosanguineum* Zucc*.* | | 1 | 1-III | | 2 | | +, – | –,+ | | L | | | | | | | + | | Suc (R), St | | | | perennial | | | 14 | | | | | | | | | |  | | | | | |
| *Tetranema roseum* (M.Martens et Galeotti) Stendl.et.Steyerm*.* | | 1 | 1-II | | 2 | | +, – | –,– | | L | | | | | | | – | | n.d. | | | | perennial | | | 2 | | | | | | | | | |  | | | | | |
| *Veronica anagallis-aquatica* L. | | 2 | 2-II | | 1 | | – | – | | L | | | | | | | + | | n.d. | | | | annual | | | 2 | | | | | | | | | |  | | | | | |
| *Veronica chamaedrys* L. | | 2 | 2-II | | 1 | | – | - | | Ch | | | | | | | + | | n.d. | | | | perennial | | | 2 | | | | | | | | | |  | | | | | |
| *Veronica fruticans Jacq.* | | 2 | 2-I | | 1 | | – | – | | Ch | | | | | | | + | | n.d. | | | | perennial | | | 2 | | | | | | | | | |  | | | | | |
| *Veronica longifolia* L. | | 1 | 1-I | | 1 | | + | – | | L | | | | | | | + | | n.d. | | | | perennial | | | 2 | | | | | | | |  | | | | | | | |
| Polemoniaceae | | | | | | | | | | | | | | | | | | | | | | | | | | | | | | | | | | | |  | | | | | |
| *Phlox paniculata* L. | | 2 | 2-I | | 1 | | – | – | | Ch | | | | | | | + | | n.d. | | | | perennial | | | | | 2 | | | | | | | |  | | | | | |
| *Polemonium sibiricum* D.Don | | 2 | 2-II | | 1 | | – | – | | Ch | | | | | | | + | | n.d. | | | | perennial | | | | | 2 | | | | | | | |  | | | | | |
| Rubiaceae | | | | | | | | | | | | | | | | | | | | | | | | | | | | | | | | | | | |  | | | | | |
| *Adina racemosa* Miq. | | 0 | 0 | | 1 | | - | – | | L | | | | | | | - | | n.d. | | | | tree | | | 12 | | | | | | | | | |  | | | | | |
| *Adina rubella* Hance. | | 1 | 1-II | | 2 | | +,- | – | | L | | | | | | | - | | n.d. | | | | shrub | | | 12 | | | | | | | | | |  | | | | | |
| *Asperula odorata* L. | | 2 | 2-III | | 1 | | – | + | | Ch | | | | | | | + | | n.d. | | | | perennial | | | 12 | | | | | | | | | |  | | | | | |
| *Canthium parviflorum* Roxb. | | 1 | 1-I | | 1 | | + | – | | L | | | | | | | – | | n.d. | | | | tree | | | 2 | | | | | | | | | |  | | | | | |
| *Catesbaea spinosa* | | 1 | 1-I | | 1 | | + | – | | L (starch) | | | | | | | - | | n.d. | | | | tree | | | 12 | | | | | | | | | | – | L (starch) | – | n.d. | tree | 12 |
| *Cephalanthus occidentalis* L. | | 1 | 1-II | | 2 | | +,- | – | | L | | | | | | | - | | n.d. | | | | tree | | | 12 | | | | | | | | | |  | | | | | |
| *Chiococca alba* (L.) Hitchc. | | 1 | 1-I | | 1 | | + | – | | L (starch) | | | | | | | – | | n.d. | | | | shrub | | | 12 | | | | | | | | | |  | | | | | |
| *Cinchona pubescens* Vahl* | | 0 | 0 | | 1 | | + | – | | L | | | | | | | – | | n.d. | | | | shrub | | | 12 | | | | | | | | | |  | | | | | |
| *Coprosma repens* A.Rich. | | 1 | 1-I(1-II) | | 1(2) | | +(+,-) | – | | L | | | | | | | – | | n.d. | | | | shrub | | | 12 | | | | | | | | | |  | | | | | |
| *Cоffea arabica* L. | | 1 | 1-I | | 1 | | + | – | | L (starch) | | | | | | | – | | St, R, Suc | | | | tree | | | 12; 18 | | | | | | | | | |  | | | | | |
| *Cruciata glabra* (L.) Ehrend. | | 2 | 2-III | | 1 | | – | + | | Ch | | | | | | | + | | n.d. | | | | perennial | | | 12 | | | | | | | | | |  | | | | | |
| *Galium aparine* L. | | 2 | 2-III | | 1 | | – | + | | Ch | | | | | | | + | | n.d. | | | | annual | | | 12 | | | | | | | | | |  | | | | | |
| *Galium boreale* L. | | 2 | 2-III | | 1 | | – | + | | Ch | | | | | | | + | | n.d. | | | | perennial | | | 12 | | | | | | | | | |  | | | | | |
| *Galium capsicum* Stev. | | 2 | 2-III | | 1 | | – | + | | Ch | | | | | | | + | | n.d. | | | | annual | | | 12 | | | | | | | | | |  | | | | | |
| *Galium krylovianum* (Serg.) Pobed. | | 2 | 2-III | | 1 | | – | + | | Ch | | | | | | | + | | n.d. | | | | annual | | | 12 | | | | | | | | | |  | | | | | |
| *Galium verum* L. | | 2 | 2-III | | 1 | | – | + | | Ch | | | | | | | + | | n.d. | | | | perennial | | | 12 | | | | | | | | | |  | | | | | |
| *Gardenia jasminoides* Ellis | | 1 | 1-I | | 1 | | + | – | | L | | | | | | | – | | n.d. | | | | shrub | | | 12 | | | | | | | | | |  | | | | | |
| *Gardenia radicans* Thunb. | | 1 | 1-I | | 1 | | + | – | | L | | | | | | | – | | n.d. | | | | shrub | | | 12 | | | | | | | | | |  | | | | | |
| *Guettarda uruguensis* Cham. et Schlecht. | | 1 | 1-I | | 1 | | + | – | | L | | | | | | | – | | n.d. | | | | tree | | | 12 | | | | | | | | | |  | | | | | |
| *Hamelia patens* Jacq. | | 1 | 1-I | | 1 | | + | – | | L | | | | | | | – | | n.d. | | | | tree | | | 12 | | | | | | | | | |  | | | | | |
| *Ixora coccinea* L. | | 1 | 1-I | | 1 | | + | – | | L | | | | | | | – | | n.d. | | | | tree | | | 12 | | | | | | | | | |  | | | | | |
| *Mitchella repens* L. | | 0 | 0 | | 1 | | + | – | | L | | | | | | | – | | n.d. | | | | perennial | | | 12 | | | | | | | | | |  | | | | | |
| *Nauclea orientalis* L. | | 1 | 1-I (1-II) | | 1(2) | | +(+,–) | – | | L | | | | | | | – | | Suc | | | | tree | | | 12 | | | | | | | | | |  | | | | | |
| *Paederia scandens* Merr. | | 1 | 1-I (1-II) | | 1(2) | | +(+,–) | – | | L | | | | | | | – | | n.d. | | | | shrub | | | 12 | | | | | | | | | |  | | | | | |
| *Pentas lanceolata* (Forssk.) Deflers | | 1 | 1-I | | 1 | | + | – | | L | | | | | | | – | | n.d. | | | | shrub | | | 12 | | | | | | | | | |  | | | | | |
| *Psychotria bacteriophila* Val. | | 1 | 1-I | | 1 | | + | – | | L (starch) | | | | | | | – | | n.d. | | | | tree | | | 2 | | | | | | | | | |  | | | | | |
| *Randia dumetorum* Lam. | | 1 | 1-I | | 1 | | + | – | | L | | | | | | | – | | n.d. | | | | shrub | | | 12 | | | | | | | | | |  | | | | | |
| *Rogiera suffrutescens* Brandegee | | 1 | 1-I | | 1 | | + | – | | L | | | | | | | – | | n.d. | | | | shrub | | | 2 | | | | | | | | | |  | | | | | |
| *Rondeletia odorata* Jacq. | | 1 | 1-I | | 1 | | + | – | | L | | | | | | | – | | n.d. | | | | shrub | | | 12 | | | | | | | | | |  | | | | | |
| *Rubia cordifolia* L. | | 2 | 2-III | | 1 | | – | + | | Ch | | | | | | | + | | n.d. | | | | perennial | | | 12 | | | | | | | | | |  | | | | | |
| *Serardia arvensis* L. | | 2 | 2-III | | 1 | | – | + | | Ch | | | | | | | + | | n.d. | | | | annual | | | 12 | | | | | | | | | |  | | | | | |
| *Uncarya rhynchophylla* (Miq.) Jacks. | | 1 | 1-I | | 1 | | + | – | | L (starch) | | | | | | | – | | n.d. | | | | tree | | | 2 | | | | | | | | | |  | | | | | |
| Scrophulariaceae | | | | | | | | | | | | | | | | | | | | | | | | | | | | | | | | | | | |  | | | | | |
| *Alonsoa meridionalis O.Kuntze* | | 1 | 1-II(1-I) | | 2 | | +, – | – | | L | | | | | | | + | | St, R, Suc | | | | semishrub | | | 13 | | | | | | | | | |  | | | | | |
| *Alonsoa warscewiczii* Regal. | | 1 | 1-II | | 2 | | +, – | – | | L | | | | | | | + | | St, R, Suc | | | | semishrub | | | 14 | | | | | | | | | |  | | | | | |
| *Buddleja albiflora* Hemsl. | | 1 | 1-I | 1 | | | + | – | | L | | | | | | | – | | | St, R, Suc (V, Ino) | | tree | | | | 2 | | | | | | | | | |  | | | | | |
| *Buddleja allemifolia* Maxim. | | 1 | 1-I | 1 | | | + | – | | L | | | | | | | – | | | St, R, Suc (V, Ino) | | tree | | | | 2 | | | | | | | | | |  | | | | | |
| *Buddleja davidii* Franch. | | 1 | 1-I | 1 | | | + | – | | L | | | | | | | – | | | St, R, Suc (V, Ino) | | tree | | | | 2 | | | | | | | | | |  | | | | | |
| *Buddleja lindleyana* | | 1 | 1-I | 1 | | | + | – | | L | | | | | | | – | | | St, R, Suc (V, Ino) | | tree | | | | 2 | | | | | | | | | |  | | | | | |
| *Buddleja salvifolia* Lam. | | 1 | 1-I | 1 | | | + | – | | L | | | | | | | – | | | St, Suc, R (V, Ino) | | tree | | | | 2 | | | | | | | | | |  | | | | | |
| *Nicodemia diversifolia* Tenore | | 1 | 1-I | 1 | | | + | – | | L | | | | | | | – | | | n.d. | | | tree | | | 2 | | | | | | | | | |  | | | | | |
| *Nemesia strumosa* Benth*.* | | 1 | 1-III | | 2 | | +,– | –,+ | | L | | | | | | | + | | Suc, St, Gol (R) | | | | annual | | | 14 | | | | | | | | | |  | | | | | |
| *Scrophularia altaica* Murr. | | 1 | 1-I | | 1 | | + | - | | L | | | | | | | – | | St, Suc | | | | perennial | | | 2 | | | | | | | | | |  | | | | | |
| *Scrophularia incica* Weinm. | | 1 | 1-I | | 1 | | + | - | | L | | | | | | | – | | St, Suc | | | | perennial | | | 2 | | | | | | | | | |  | | | | | |
| *Scrophularia americana* Bordz*.* | | 1 | 1-I(1-II) | | 1(2) | | +(+,–) | – | | L | | | | | | | – | | n.d. | | | | perennial | | | 2 | | | | | | | | | |  | | | | | |
| *Verbascum chaixi* Vill*.* | | 1 | 1-I | | 1 | | +, | – | | L | | | | | | | – | | Suc, R, St | | | | perennial | | | 14 | | | | | | | | | |  | | | | | |
| *Verbascum ovalifolium* Donn ex Sims | | 1 | 1-II | | 2 | | +, – | –,– | | L | | | | | | | – | | n.d. | | | | perennial | | | 2 | | | | | | | | | |  | | | | | |
| Solanaceae | | | | | | | | | | | | | | | | | | | | | | | | | | | | | | | | | | | |  | | | | | |
| *Atropa bella-donna*L. | | 2 | 2-IV | | 1 | | – | + | Ch | | | | | | | | + | | n.d. | | | | shrub | | | 2 | | | | | | | | | |  | | | | | |
| *Brunfelsia pauciflora* (Cham. et Schlecht) Benth. cv. Exima | | 2 | 2-I | | 1 | | – | – | Ch | | | | | | | | + | | n.d. | | | | shrub | | | 2 | | | | | | | | | |  | | | | | |
| *Cestrum parqui* L'Her. | | 2 | 2-III | | 1 | | – | + | Ch | | | | | | | | + | | n.d. | | | | shrub | | | 2 | | | | | | | | | |  | | | | | |
| *Datura arborea* L. | | 2 | 2-I | | 1 | | – | – | Ch | | | | | | | | + | | Suc | | | | shrub | | | 2 | | | | | | | | | |  | | | | | |
| *Hyoscyamus* *albus* L. | | 2 | 2-I | | 1 | | – | – | Ch | | | | | | | | + | | n.d. | | | | perennial | | | 2 | | | | | | | | | |  | | | | | |
| *Lycium ruthenicum* Murr. | | 2 | 2-III | | 1 | | – | + | Ch | | | | | | | | + | | n.d. | | | | shrub | | | 2 | | | | | | | | | |  | | | | | |
| *Lycopersicon esculentum* Mill. | | 2 | 2-I | | 1 | | – | + | Ch | | | | | | | | + | | n.d. | | | | perennial | | | 2 | | | | | | | | | |  | | | | | |
| *Nicotiana benthamiana*  L. | | 2 | 2-I | | 1 | | – | – | Ch | | | | | | | | + | | n.d. | | | | perennial | | | 15 | | | | | | | | | |  | | | | | |
| *Nicotiana tabacum* L. | | 2 | 2-I | | 1 | | – | – | Ch | | | | | | | | + | | n.d. | | | | perennial | | | 16 | | | | | | | | | |  | | | | | |
| *Physochlaina* *orientalis* (Bieb.) G.Don | | 2 | 2-III | | 1 | | – | + | Ch | | | | | | | | + | | n.d. | | | | annual | | | 2 | | | | | | | | | |  | | | | | |
| *Scopolia stramonifolia* (Wall.) Sem. | | 2 | 2-I | | 1 | | – | – | Ch | | | | | | | | + | | n.d. | | | | perennial | | | 2 | | | | | | | | | |  | | | | | |
| *Solanum aviculare* Forst. | | 2 | 2-I | | 1 | | – | – | Ch | | | | | | | | + | | n.d. | | | | perennial | | | 2 | | | | | | | | | |  | | | | | |
| *Solanum caniarenze* Juz. et Buc. | | 2 | 2-I | | 1 | | – | – | Ch | | | | | | | | + | | n.d. | | | | perennial | | | 2 | | | | | | | | | |  | | | | | |
| *Solanum dulcamara* L. | | 2 | 2-I | | 1 | | – | – | Ch | | | | | | | | + | | n.d. | | | | perennial | | | 2 | | | | | | | | | |  | | | | | |
| *Solanum giganteum* Jacq. | | 2 | 2-I | | 1 | | – | – | Ch | | | | | | | | + | | Suc | | | | perennial | | | 2 | | | | | | | | | |  | | | | | |
| *Solanum melongena* L. | | 2 | 2-I | | 1 | | – | – | Ch | | | | | | | | + | | n.d. | | | | perennial | | | 2 | | | | | | | | | |  | | | | | |
| *Solanum rantonnetii* Carr. | | 2 | 2-I | | 1 | | – | – | Ch | | | | | | | | + | | n.d. | | | | perennial | | | 2 | | | | | | | | | |  | | | | | |
| *Solanum tuberosum* L. | | 2 | 2-I | | 1 | | – | – | Ch | | | | | | | | + | | n.d. | | | | perennial | | | 17 | | | | | | | | | |  | | | | | |
| Verbenaceae | | | | | | | | | | | | | | | | | | | | | | | | | | | | | | | | | | | |  | | | | | |
| *Duranta erecta* L. | | 1 | 1-I | | 1 | | + | – | L | | | | | | | | – | | n.d. | | | | shrub | | | 2 | | | | | | | | | |  | | | | | |
| *Lantana camara*L. | | 1 | 1-II | | 2 | | +,– | – | L | | | | | | | | – | | n.d. | | | | tree | | | 2 | | | | | | | | | |  | | | | | |
|  | *minor veins in cortex were studied  ** Xylem and phloem are separated by a lactifier cell  *** Sugars analyzed in leaf extracts but no data on phloem sap composition are available | | | | | | | | | | | | | | | | | | | | | | | | | | | | | |  | | | | | | | | | | |
